# Soil weathering dynamics and erosion in a dry oceanic area of the southern hemisphere (Otago, New Zealand)

**DOI:** 10.1038/s41598-022-23731-7

**Published:** 2022-11-17

**Authors:** Gerald Raab, Markus Egli, Kevin P. Norton, Adam P. Martin, Michael E. Ketterer, Dmitry Tikhomirov, Rahel Wanner, Fabio Scarciglia

**Affiliations:** 1 Department of Geography, University of Zurich, Winterthurerstrasse 190, 8057 Zurich, Switzerland; 2 Department of Earth and Environmental Sciences, Dalhousie University, Oxford Street, PO BOX 15000, Halifax, 1459 Canada; 3 School of Geography, Environment and Earth Sciences, Te Herenga Waka, Victoria University of Wellington, PO Box 600, Wellington, 6140 New Zealand; 4 GNS Science, Private Bag 1930, Dunedin, New Zealand; 5 Chemistry and Biochemistry, Northern Arizona University, Box 5698, Flagstaff, AZ 86011-5698 USA; 6 Institute of Natural Resource Sciences, Zurich University of Applied Sciences, Grüental, 8820 Wädenswil, Switzerland; 7 Department of Biology, Ecology and Earth Sciences (DiBEST), University of Calabria, Via P. Bucci-Cubo 15B, 87036 Arcavacata Di Rende, CS Italy

**Keywords:** Geochemistry, Geomorphology

## Abstract

Landscape evolution is driven by tectonics, climate and surface denudation. In New Zealand, tectonics and steep climatic gradients cause a dynamic landscape with intense chemical weathering, rapid soil formation, and high soil losses. In this study, soil, and elemental redistribution along two adjacent hillslopes in East Otago, New Zealand, having different landscape settings (ridge versus valley) are compared to identify soil weathering and erosion dynamics. Fallout radionuclides (^239+240^Pu) show that over the last ~ 60 years, average soil erosion rates in the valley (~ 260 [t km^−2^ year^−1^]) are low compared to the ridge (~ 990 [t km^−2^ year^−1^]). The ridge yields up to 26% lower soil weathering intensity than the topographical-protected valley. The lowest soil weathering intensity is found at both hilltop positions, where tors (residual rocks) are present and partially disintegrate. The soil weathering intensity increases with distance from tors, suggesting that tors rejuvenate the chemical weathering signature at the hilltop positions with fresh material. The inversed and decreasing weathering degree with all soil depth indicates that the fresh mineral contribution must be higher at the soil surface than at the bedrock weathering front. Higher erosion rates at the exposed ridge may be partially attributed to wind, consistent with rock abrasion of tors, and low local river sediment yields (56 [t km^−2^ year^−1^]). Thus, the East Otago spatial patterns of soil chemistry and erosion are governed by tor degradation and topographic exposure.

## Introduction

**Figure 1:**
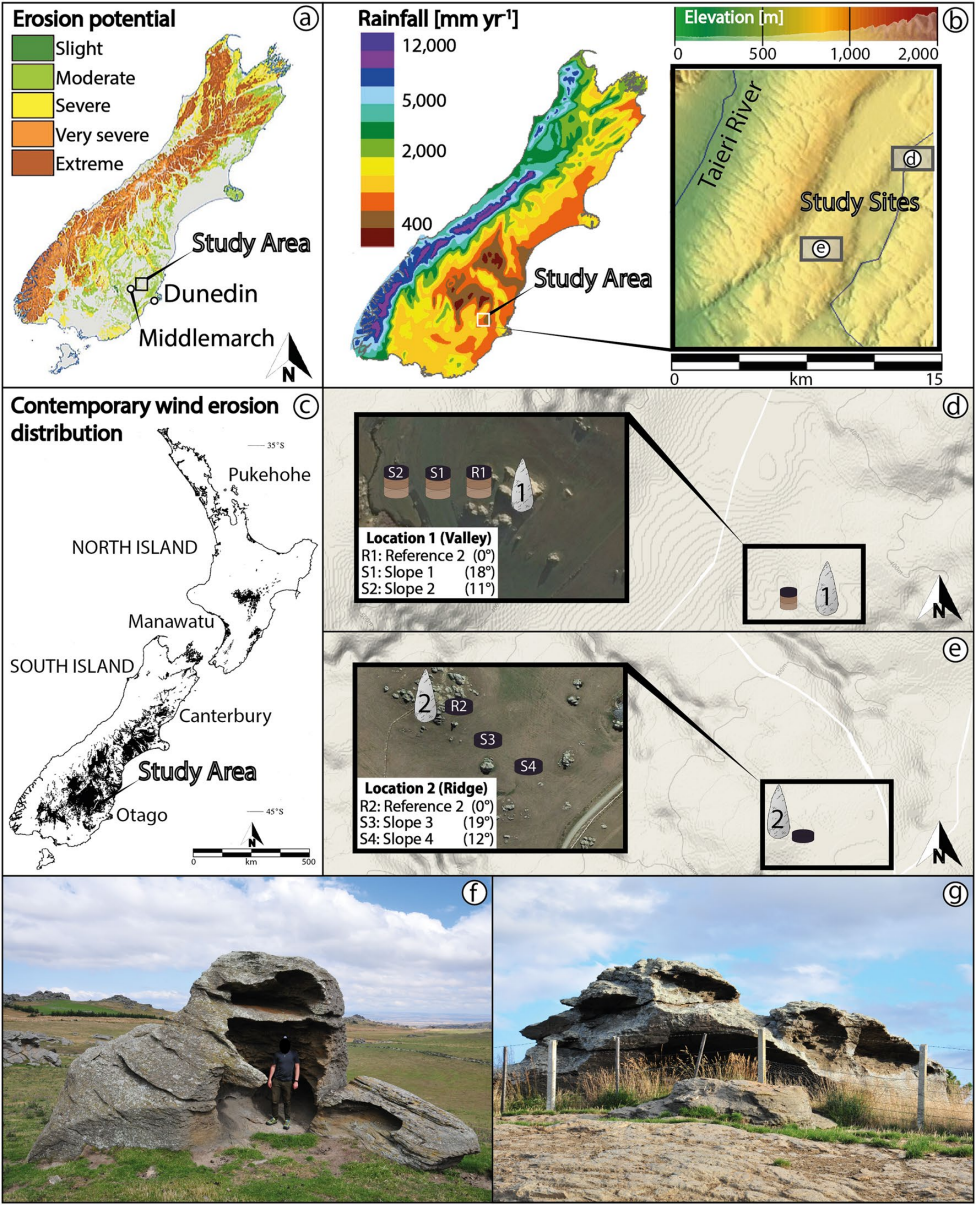
(**a**) Potential soil erosion map of New Zealand ‘s South Island^7^. (**b**) Annual rainfall of New Zealand’s South Island^8^ and position of the study area with study site locations. (**c**) Map of the contemporary wind erosion distribution (darkened areas represent the contemporary wind erosion distribution) in New Zealand from the New Zealand Land Resource Inventory^9^. (**d**,**e**) detailed study site sampling maps (Google Maps and Google Earth, 2019). Both (**f**) and (**g**) are examples of highly weathered and hollowed out local schist tors at the investigated ridge.

New Zealand loses about 200 [Mt year^−1^] of sediment through erosion to the ocean^1^, discharging about 1–2% of the world's annual average of sediment to the ocean while making up only ~ 0.1% of the global land mass^2^. Currently, one-third of New Zealand is prone to wind erosion^3,4^, which is particularly prevalent in the east of the South Island. The exposed uplands of Otago province (Fig. 1a), with their depleted grassland vegetation and semi-arid environment (Fig. 1b), are particularly affected by wind erosion^5^. Weathering intensity data on the west coast of the South Island indicated that soil chemical denudation rates increase proportionally with erosion rates among the highest levels observed globally^6^, but the weathering dynamics are poorly constrained. Previous work showed that soil is produced more rapidly from bedrock than previously recognized and that the denudation rates primarily control the pace of these high soil production rates.

In general, chemical weathering and physical erosion rates increase with higher precipitation. In contrast, high vegetation cover decreases physical erosion rates and thus also denudation and soil production rates^10^, displaying a co-depends between vegetation growth and soil stabilization rates. Active tectonic processes and steep climatic gradients in New Zealand lead to a highly dynamic landscape with intense chemical weathering, high soil formation rates, and high soil losses. The complex interplay between these processes is only starting to be investigated in the dynamic New Zealand environment. Several mapping campaigns to study erosion have been undertaken^4,5,11^. Among these, soil-surface dynamic studies have used ^137^Cs as the quantitative soil erosion tracer^12–14^. However, the fallout radionuclides (FRNs) ^239+240^Pu are increasingly used for quantifying soil erosion rates relative to ^137^Cs, due to the advantages of a longer half-life, often lower spatial variability, and lower analytical uncertainty^15,16^.

In this study, soil, and element redistributions of two transects in contrasting semi-arid landscape settings of a valley and an adjacent ridge in East Otago, South Island, New Zealand (Fig. 1a), are investigated to better understand weathering and erosion dynamics in rocky dry landscapes. We used a multi-method approach that integrates soil geochemistry, weathering indices, carbon isotopes and fallout radionuclides along the two transects. We show how major and trace elements (and consequently chemical weathering indices) differ between the two landscape settings; how they compare to erosion rates in other parts of New Zealand and other (semi-)arid areas globally; how the results using the ^239+240^Pu-based method compare to previous results using ^137^Cs as an erosion tracer; and how soil redistribution rates (erosion and deposition) correlate with chemical weathering patterns as a function of topography.

## Results

### General site and soil properties

The investigated sites are east of the hilly Otago upland (12°–25° slope angle; Fig. 1a,b,d,e; Table 1) above 300 masl. Two hillslopes in close proximity (~ 5 km; Fig. 1d,e) but with differing landscape settings (Fig. 1b) were sampled, one in a valley (location 1; Fig. 1d) and the other on an exposed ridge (location 2; Fig. 1e; Table 1; see “Materials and methods” for more details). The studied soils are weakly developed and show limited signs of soil-forming processes. Both study sites are classified as laminar pallic soils in the Soil Map of New Zealand^17^. They are silty, seasonally dry (dry in summer and wet in winter), with pale-coloured (light yellow–brown to olive yellow), high-density subsoils which are low in iron oxides (in comparison to other New Zealand soil orders), and poorly structured^18^. Field observations instead suggest a Regosols and Leptosols per the World Reference Base (WRB) classification^19^ (Table 1). A topsoil with distinct humus accumulation is present (Table 2), but underlying B horizons are weakly expressed and highly mixed with angular schist rock clasts, which in places constitute definite gravel layers. The profile differentiation among all soil pits is weak, with a shallow A (< 15 cm) and B_w_ (< 20 cm) horizon in places separated by wavy/lobate or irregular boundaries (S1, S2, S3, S4 sites).

**Table 1:** Study site characteristics.

Site	Coordinates (WGS 84)		Elevation (m a.s.l.)	Slope	Parent material	Vegetation	WRB soil orders
**Location 1 (Valley)**							
Reference (R1)	S 45° 27.180′	E 170° 20.660′	374	0°	Otago Schist	Pasture	Leptosol
Back slope 1 (S1)	S 45° 27.173′	E 170° 20.636′	370	18°	Otago Schist	Pasture	Regosol
Foot slope 2 (S2)	S 45° 27.174′	E 170° 10.614′	363	11°	Otago Schist	Pasture	Regosol
**Location 2 (Ridge)**							
Reference (R2)	S 45° 29.968′	E 170° 16.660′	582	0°	Otago Schist	Pasture	Leptosol
Back slope 1 (S3)	S 45° 29.977′	E 170° 16.643′	578	19°	Otago Schist	Pasture	Regosol
Foot slope 2 (S4)	S 45° 29.994′	E 170° 16.660′	573	12°	Otago Schist	Pasture	Leptosol

**Table 2:** Physical and chemical characteristics of the investigated soils.

	Depth (cm)	Bulk density (g cm^−3^)	Soil skeleton (%)	pH (–)	LOI^a^ (%)	N (g kg^−1^)	C_tot_ (g kg^−1^)	OM^b^ (g kg^−1^)	C/N (–)	δ^13^C (‰)
Location 1 (Valley)				(CaCl_2_)	(550 °C)					
**Reference site (R1)**										
^c^L1-R1-P1-1	0–5	0.99	11.8	4.69	19.3	7.18	81.90	141.2	11.4	− 25.01
	5–10	0.86	6.3	4.42	12.2	5.24	46.50	80.2	8.9	− 24.75
	10–20	1.75	35.7	4.02	8.6	3.32	28.90	49.8	8.7	− 25.38
	20–25	1.78	56.4	3.94	7.1	2.96	21.90	37.8	7.4	− 24.80
L1-R1-P1-2	0–5	1.10	22.8	4.92	20.4	7.27	87.70	151.2	12.1	− 24.93
	5–10	1.30	38.5	4.5	13.0	5.64	50.30	86.7	8.9	− 25.18
	10–20	1.85	52.1	4.37	9.8	3.86	35.30	60.9	9.1	− 26.61
	20–25	1.75	58.4	4.06	7.3	2.85	22.60	39.0	7.9	− 26.10
L1-R1-P2-1	0–5	1.07	12.9	4.48	22.4	8.02	98.10	169.2	12.2	− 25.69
	5–10	1.64	21.1	4.08	10.5	4.15	39.30	67.8	9.5	− 25.50
	10–20	2.32	60.0	3.87	7.8	3.99	25.20	43.5	6.3	− 24.36
	20–25	2.40	66.6	3.92	6.2	3.05	18.20	31.4	6.0	− 27.67
L1-R1-P2-2	0–5	0.92	9.7	4.52	24.0	7.96	106.00	182.8	13.3	− 27.32
	5–10	1.64	24.1	4.13	11.5	4.55	44.70	77.1	9.8	− 26.66
	10–20	2.26	63.2	4.03	7.7	2.28	26.40	45.5	11.6	− 24.97
	20–25	2.30	67.5	4.03	6.9	3.12	21.80	37.6	7.0	− 26.87
**Slope 1 (S1)**										
L1-S1-P1-1	0–5	1.32	25.7	4.45	18.3	6.36	75.30	129.9	11.8	− 28.05
	5–10	1.49	38.6	4.27	12.0	4.37	45.60	78.6	10.4	− 27.67
	10–20	1.89	54.6	4.29	10.4	4.29	34.60	59.7	8.1	− 27.33
	20–30	1.89	44.4	4.25	8.4	3.48	25.40	43.8	7.3	− 26.70
	30–40	2.08	36.2	4.29	6.2	2.46	12.10	20.9	4.9	− 28.24
L1-S1-P1-2	0–5	1.16	36.0	4.60	16.2	6.9	83.50	144.0	12.1	− 26.04
	5–10	1.45	42.8	4.36	12.5	4.21	47.40	81.7	11.3	− 26.80
	10–20	1.86	45.3	4.13	8.3	3.05	25.70	44.3	8.4	− 25.96
	20–30	1.88	27.7	4.18	7.2	2.59	17.30	29.8	6.7	− 26.22
	30–40	1.86	36.4	4.38	6.2	1.38	11.50	19.8	8.4	− 24.65
L1-S1-P2-1	0–5	1.17	20.6	4.63	18.2	7.97	76.80	132.4	9.6	− 25.56
	5–10	1.12	39.1	4.28	13.8	5	53.80	92.8	10.8	− 24.73
	10–20	1.85	50.3	4.19	10.3	2.11	36.60	63.1	17.3	− 24.77
	20–30	1.97	38.4	4.28	7.8	3.57	22.80	39.3	6.4	− 25.55
	30–40	2.12	38.2	4.21	5.6	1.93	11.90	20.5	6.2	− 26.77
L1-S1-P2-2	0–5	1.05	22.2	4.97	22.5	7.93	97.20	167.6	12.3	− 26.29
	5–10	1.44	49.4	4.86	15.5	6.1	62.10	107.1	10.2	− 26.77
	10–20	1.8	64.3	4.84	10.3	3.28	36.10	62.3	11.0	− 26.30
	20–30	1.88	63.6	4.94	12.4	5.12	47.10	81.2	9.2	− 25.48
	30–40	1.61	49.8	4.52	6.5	2.76	16.30	28.1	5.9	− 25.97
**Slope 2 (S2)**										
L1-S2-P1-1	0–5	0.96	25.1	5.28	13.1	4.49	52.40	90.4	11.7	− 25.98
	5–10	1.97	44.4	5.11	10.1	3.48	36.20	62.4	10.4	− 27.42
	10–20	1.85	61.4	4.71	11.8	5.39	45.00	77.6	8.4	− 26.96
	20–30	2.17	49.4	4.27	5.9	2.68	17.20	29.7	6.4	− 26.34
	30–40	2.52	73.9	4.18	3.9	1.77	6.99	12.1	4.0	− 25.61
L1-S2-P1-2	0–5	1.44	24.9	5.47	11.6	5.36	44.00	75.9	8.2	− 27.21
	5–10	1.23	33.8	4.91	10.1	4.67	37.10	64.0	8.0	− 27.01
	10–20	1.67	67.9	4.84	11.3	4.79	42.90	74.0	9.0	− 27.12
	20–30	2.09	50.4	4.23	5.3	2	13.70	23.6	6.8	− 26.47
	30–40	2.62	77.8	4.12	3.6	2.02	5.39	9.3	2.7	− 26.13
L1-S2-P2-1	0–5	1.32	29.4	5.08	11.1	4.63	40.70	70.2	8.8	− 27.18
	5–10	1.49	42.7	4.82	11.3	4.59	41.50	71.6	9.1	− 27.05
	10–20	1.89	54.1	4.50	9.7	4.04	34.80	60.0	8.6	− 27.02
	20–30	1.89	63.3	4.29	5.0	2.29	11.70	20.2	5.1	− 25.22
	30–40	2.08	74.6	4.18	2.9	2.25	3.85	6.6	1.7	− 25.91
L1-S2-P2-2	0–5	1.32	29.8	4.91	10.6	4.15	39.40	67.9	9.5	− 27.10
	5–10	1.49	32.6	4.73	10.8	4.32	40.10	69.2	9.3	− 26.98
	10–20	1.89	52.6	4.23	6.2	2.78	17.50	30.2	6.3	− 26.4
	20–30	1.89	54.7	4.12	3.6	2.56	5.24	9.0	2.1	− 26.27
	30–40	2.08	78.0	4.17	3.1	1.41	0.23	0.4	0.2	− 26.75
**Location 2 (Ridge)**										
Reference site (R2)										
L2-R2-P1-1	0–5	0.48	20.4	5.34	41.8	22.22	230.00	396.6	10.4	− 25.78
	5–10	0.54	7.4	4.94	34.2	17.45	194.00	334.5	11.1	− 23.93
	10–20	1.11	18.0	4.64	24.9	10.64	113.00	194.9	10.6	− 26.24
	20–30	1.29	7.0	3.94	15.0	5.9	58.20	100.4	9.9	− 25.88
L2-R2-P1-2	0–5	0.57	14.2	4.69	42.4	18.54	226.00	389.7	12.2	− 25.99
	5–10	0.91	14.8	4.08	33.2	12.26	156.00	269.0	12.7	− 26.17
	10–20	0.90	30.9	3.76	23.1	7.57	100.00	172.4	13.2	− 25.25
	20–30	1.61	9.5	4.21	11.5	4.72	38.30	66.0	8.1	− 24.14
L2-R2-P2-1	0–5	1.03	16.7	5.34	31.3	13.47	163.00	281.1	12.1	− 25.19
	5–10	1.32	14.7	4.32	19.9	5.95	90.40	155.9	15.2	− 25.00
	10–20	1.61	38.4	3.91	12.1	4.33	50.50	87.1	11.7	− 25.42
	20–30	1.65	22.4	3.64	10.1	3.07	36.60	63.1	11.9	− 26.78
L2-R2-P2-2	0–5	0.97	34.8	4.99	37.8	19.01	211.00	363.9	11.1	− 25.88
	5–10	1.25	58.0	4.20	22.6	10.59	104.00	179.3	9.8	− 24.65
	10–20	1.90	57.2	3.83	12.4	4.65	50.50	87.1	10.9	− 25.77
	20–30	1.49	34.3	3.75	10.4	4.25	39.30	67.8	9.3	− 25.79
Slope 3 (S3)										
L2-S3-P1-1	0–5	0.61	20.7	4.95	33.9	15.06	159.00	274.2	10.6	− 26.71
	5–10	1.16	17.0	3.87	19.4	6.77	81.60	140.7	12.1	− 26.25
	10–20	2.14	59.2	3.75	10.6	3.39	34.90	60.2	10.3	− 25.72
	20–30	2.17	55.9	3.78	7.5	2.28	20.40	35.2	9.0	− 25.05
	30–40	1.89	30.7	3.80	7.6	2.02	18.60	32.1	9.2	− 26.60
L2-S3-P1-2	0–5	0.74	18.4	4.89	35.2	16.44	186.00	320.7	11.3	− 25.63
	5–10	1.29	19.2	4.40	18.3	5.61	77.00	132.8	13.7	− 25.38
	10–20	1.66	47.3	3.72	11.4	4.43	39.60	68.3	8.9	− 25.26
	20–30	2.30	65.2	3.68	6.6	3.12	16.60	28.6	5.3	− 25.62
	30–40	1.85	54.8	3.69	7.1	3.24	18.60	32.1	5.8	− 24.21
L2-S3-P2-1	0–5	0.96	29.4	4.70	27.5	10.44	123.00	212.1	11.8	− 24.76
	5–10	1.59	22.2	3.97	15.6	5.59	60.20	103.8	10.8	− 25.91
	10–20	1.62	36.7	3.83	11.9	4.41	41.00	70.7	9.3	− 25.59
	20–30	1.54	49.1	3.83	9.5	3.31	29.30	50.5	8.9	− 24.77
	30–40	–	–	–	–	–	–	–	–	–
L2-S3-P2-2	0–5	0.70	13.8	4.73	36.1	14.94	178.00	306.9	11.9	− 25.46
	5–10	1.03	15.2	3.96	18.0	6.15	72.10	124.3	11.7	− 24.05
	10–20	1.67	33.0	3.83	11.8	4.44	41.20	71.1	9.3	− 24.14
	20–30	2.06	63.9	3.86	9.9	4.03	32.40	55.9	8.0	− 26.67
	30–40	–	–	–	–	–	–	–	–	–
Slope 4 (S4)										
L2-S4-P1-1	0–5	0.76	25.3	5.45	24.8	9.19	110.00	189.7	12.0	− 26.11
	5–10	1.01	50.4	5.24	17.5	7.06	72.90	125.7	10.3	− 25.47
	10–20	2.13	52.9	3.96	8.3	3.89	25.60	44.1	6.6	− 26.52
	20–30	–	–	–	–	–	–	–	–	–
L2-S4-P1-2	0–5	1.11	36.2	5.42	24.6	9.34	110.00	189.7	11.8	− 25.57
	5–10	1.16	52.7	4.97	18.3	6.59	75.70	130.5	11.5	− 24.13
	10–20	2.42	64.3	3.96	8.4	3.33	26.00	44.8	7.8	− 25.61
	20–30	–	–	–	–	–	–	–	–	–
L2-S4-P2-1	0–5	1.23	31.4	5.37	24.0	8.32	104.00	179.3	12.5	− 25.98
	5–10	1.53	48.2	5.14	17.1	6.14	70.20	121.1	11.4	− 26.39
	10–20	2.40	70.2	4.09	8.5	3.27	27.80	47.9	8.5	− 25.52
	20–30	–	–	–	–	–	–	–	–	–
L2-S4-P2-2	0–5	1.56	40.7	5.32	21.6	7.95	93.00	160.4	11.7	− 25.08
	5–10	1.42	57.5	4.76	14.1	5.58	55.60	95.9	10.0	− 26.31
	10–20	2.55	71.5	3.92	8.0	3.6	24.20	41.7	6.7	− 25.49
	20–30	–	–	–	–	–	–	–	–	–

^a^LOI (loss on ignition) = based on weight percent loss of ashed material during pre-treatment for Pu analysis.

^b^OM (organic matter) = total organic C (C_tot_) × 1.72; C_tot_ was determined by a CHN analyzer.

^c^L1 = Location 1 (L2 = Location 2).

### Physical and chemical soil characteristics

The reference sites at both landscape settings reached no more than 30 cm soil thickness (above the R horizon), while the nearby slope sites allowed the sampling of an additional 10 cm. The soil is strongly mixed with schist rock clasts of various sizes resulting in high soil density when in some cases, approaches that of underlying Otago Schist rock density (e.g., 2.65–2.78 [g cm^−3^]^20^). The bulk density of the samples at both sites increased with depth and ranged in the valley between 0.86–2.62 [g cm^−3^] and at the ridge from 0.48 [g cm^−3^] to 2.55 [g cm^−3^] (Table 2; Supplementary Fig. S1). The bulk density average is 1.69 [g cm^−3^] and 1.39 [g cm^−3^] for the valley and ridge.

The pH indicates strongly to slightly acidic conditions. It decreases with soil depth (valley: p = 0.52; ridge: p = 0.75) with higher pH values at the valley [maximum: 5.47; minimum: 3.87; average (n = 56): 4.45] compared to the ridge [maximum: 5.45; minimum: 3.64; average (n = 46): 4.35] (Table 2), yet with an homogeneity of variance (valley: ridge is F: 0.52 to F_α_: 0.63). No carbonate was present in the soils in the local quartz-rich Otago schist bedrock environment and a soil pH of around 5 or lower at all soil profiles (Table 2). In contrast, the nitrogen content is found to be higher at the ridge, with a maximum of 22.2 [g kg^−1^] compared to the valley maximum of 8.0 [g kg^−1^] (Table 2; Supplementary Fig. S1). This is also supported by the heterogeneity of variance (valley: ridge is F: 0.12 to F_α_: 0.63). The difference in the nitrogen content is most expressed within the first 5 cm of the soil, where the ridge average at 13.7 [g kg^−1^] is more than double the valley average of 6.5 [g kg^−1^] (Table 2). The nitrogen decreases mostly gradually with soil depth (valley: p = 0.80; ridge: p = 0.68) with some exceptions like C_org_ trends (see below) at both sites down to values as low as 1.4 [g kg^−1^] in the valley and 2.0 [g kg^−1^] at the ridge. The sulphur content is ~ 10 times higher in soils (Supplementary Table S2; Supplementary Fig. S1) than in adjacent rock tors (Supplementary Table S5).

The total organic carbon (C_tot_) that equals C_org_ (acidic soils and thus no carbonates present) and organic matter content present a similar yet weaker depth pattern to nitrogen (valley: p = 0.77; ridge p = 0.55). At the pits P1 and P2 at site S2 and pit P2 at site S1 (valley), the decreasing trend downwards is interrupted by a relative peak of increase at depths of 5–10 or 10–20 cm. Similarly, in pit P1 at site S3 (exposed ridge), a relative minimum occurs at a depth of 20–30 cm. This behavior with depth largely parallels the nitrogen patterns (N:C_tot_, valley: p = 0.96; ridge: p = 99). The C_tot_ of valley soils ranges from 0.23 to 106 [g kg^−1^] and ridge soils span from 16.6 to 230 [g kg^−1^]. Like nitrogen, the most considerable differences in soil C_tot_ between the two settings occur within the top 5 cm (Table 2; Supplementary Fig. S1). The average top 5 cm exhibit a C_tot_ of 72.8 [g kg^−1^] in the valley, which is only half of the C_tot_ average of 157.8 [g kg^−1^] at the ridge. The similarities of the carbon and nitrogen patterns result in similar C/N ratios (e.g., R1: R2 is F: 1.71 to F_α_: 2.40) among the two landscape settings. However, in pit P2 at site R2 (exposed ridge), the parallel pattern is interrupted at a depth of 10–20 cm.

### Carbon isotopes (δ^13^C)

**Figure 2:**
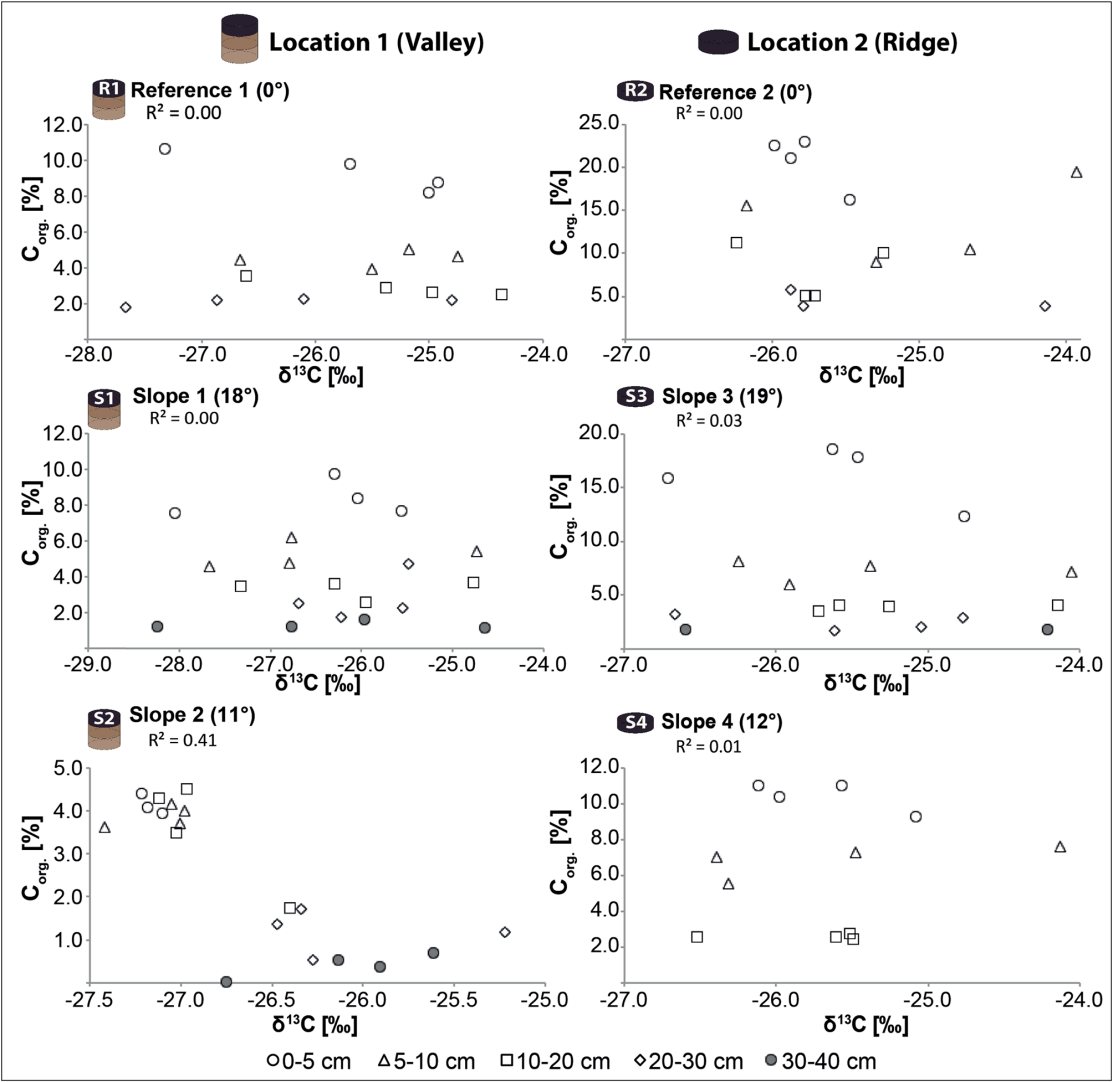
Correlation between C_org_ (organic carbon content) and δ^13^C values as an indicator of soil disturbance/stability (the values are given in Table 2).

The δ^13^C values range between − 28.2‰ and − 24.4‰ in the valley and span from − 26.8 to − 23.9 ‰ at the ridge (Table 2; Fig. 2; Supplementary Fig. S2). The δ^13^C-signature is within the range of C3-grasses (− 20 to − 30‰) and is typical for cool and dry areas. The anticipated negative correlation (see “Determination of short- and mid-term soil erosion”) between δ^13^C and C_org_ was not encountered, despite the logarithmic decrease of C_org_ with soil depth at most sites (Table 2; Fig. 2, Supplementary Fig. S2), which we attribute to the large variability in δ^13^C. Field observations did not indicate any presence of carbonates that could impact the signal. Also, a presence of inorganic C has not been detected since the loss on ignition differences between soil ashing for XRF (1000–1050 °C) and Pu isotope investigations (550 °C) are within the natural error range (~ 1.9%).

### Soil redistribution rates: ^239+240^Pu

**Figure 3:**
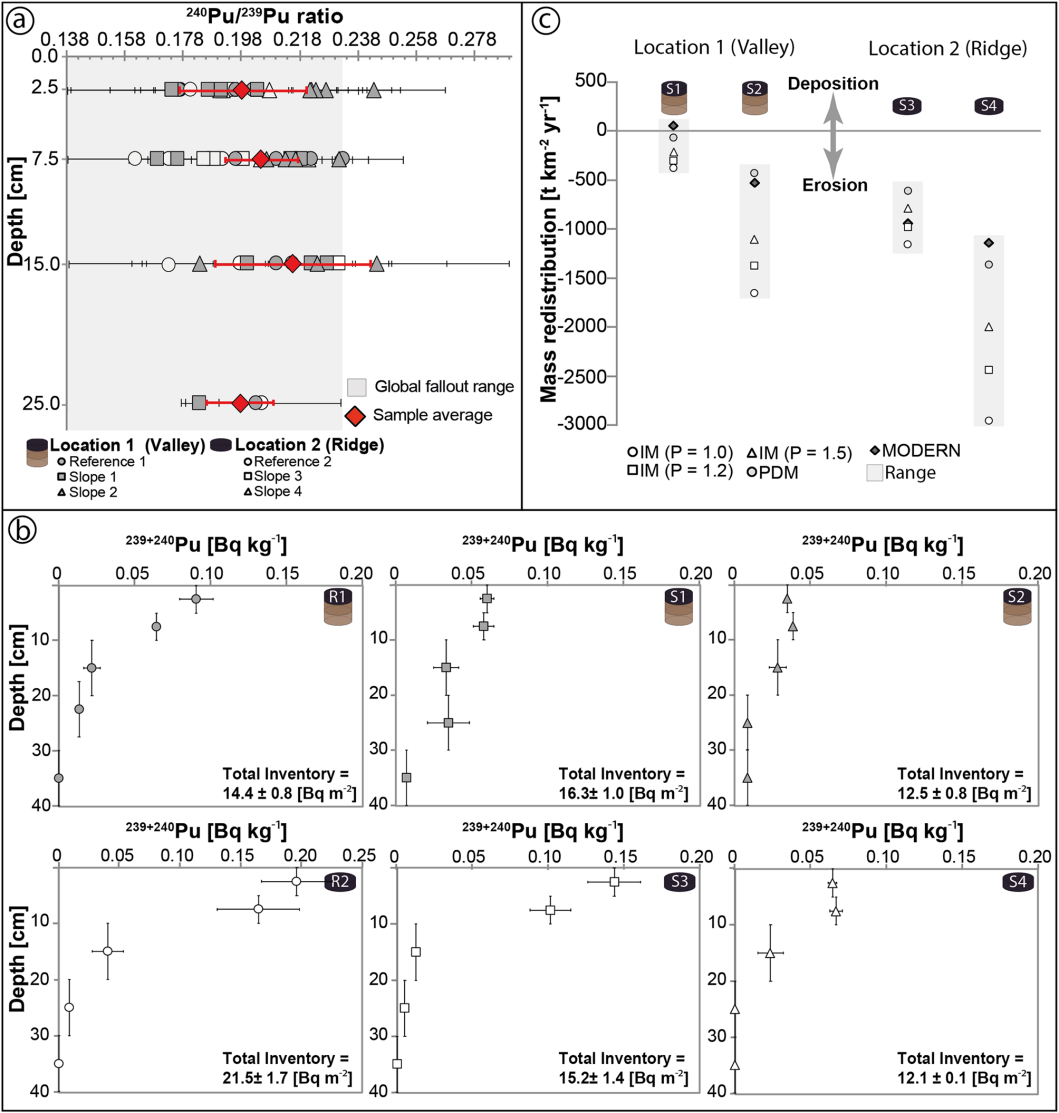
(**a**) The ^240^Pu/^239^Pu ratios and standard error of the soil samples as a function of soil depth. The average for each depth increment is given in red. The grey area indicates the global fallout range (0.185 ± 0.047) of the southern hemisphere^21^. (**b**) Depth-activity profiles (± standard error) of the investigated sites. (**c**) Calculated annual average soil redistribution for both reference sites using different particle size correction factors (P = 1.0, 1.2, 1.5) for the inventory method^22^, together with results of the profile distribution model^23,24^. Detailed values of the individual soil profiles of each pit are given in Supplementary Table S0.

Nine samples (20–40 cm depth) were below the detection limit (< 0.002 [Bq kg^−1^]). Sites at the valley and the ridge both have similar, and consistent ^240^Pu/^239^Pu ratios along their depth increments (Fig. 3a). Insignificantly higher ratios were measured in the valley at reference site (R) 1 (0.233 ± 0.003) and in two samples from the slope (S) 2 site (0.244 ± 0.009; 0.245 ± 0.024). The lowest ratios were found at R2 (0.160 ± 0.032) and S3 (0.164 ± 0.026). Overall, the ^239+240^Pu activity distribution (Fig. 3b) along both reference profiles and S3 exponentially decreases with depth. Profiles of S1, S2, and S4, where the exponential trend is less regular, show equal activity within the top 10 cm. Both near-gully sites S2 and S4 have a similar total ^239+240^Pu inventory (Fig. 3b). In the valley, the total inventory increases to the intermediate slope (S1) and decreases at the slope foot (S2) (Fig. 3b). On the ridge, the total inventory decreases continuously from the top to the foot of the hill. Overall, the ridge has, on average, ~ 2.5-fold higher erosion rates (Fig. 3c) than the valley. Both sites reflect similar trends of the total Pu inventory (Fig. 3b) from hilltop to hill foot. The inventory increases from the reference sites at the top of the hills (R1: 14.4 [Bq m^−2^]; R2: ~ 21.5 [Bq m^−2^]), to the backslope (S1: ~ 16.3 [Bq m^−2^]; S3: ~ 15.2 [Bq m^−2^]) and decreases at the foot slope of the hill (S2: ~ 12.5 [Bq m^−2^], S4: ~ 12.1 [Bq m^−2^]). The highest inventory was measured at R2 [21.5 ± 1.7 Bq m^−2^].

We used three different approaches to interpret fallout nuclide inventories (see “Determination of short- and mid-term soil erosion”): the inventory method (IM), the profile distribution model (PDM), and MODERN (Modelling Deposition and Erosion rates with RadioNuclides). The IM yielded average erosion rates of valley soil sites between 664 [t km^−2^ year^−1^] and 996 [t km^−2^ year^−1^]. At the ridge, the overall average erosion rates are about 1376 [t km^−2^ year^−1^] to 2064 [t km^−2^ year^−1^] (IM, Supplementary Table S0). The highest average erosion rates occurred at the hill foot sites S4 (~ 2922 [t km^−2^ year^−1^]) and S2 (1652 [t km^−2^ year^−1^]) when using the IM. These are extremely high values. The PDM yielded the lowest average soil erosion rates at 86 [t km^−2^ year^−1^] for S1 and ~ 435 [t km^−2^ year^−1^] for S4. The mass redistribution rates calculated by the IM were up to three times higher compared to the PDM. Both models resulted in a narrower soil redistribution range at the intermediate slopes S1 and S3. Overall, the soil erosion rates at back slope positions (S1, S3) are lower than the foot slopes (S2, S4) as the latter is near an adjacent gully. In contrast to all other models, soil accumulation could be calculated for site S2 using MODERN. The PDM model and MODERN soil redistribution rates were similar (Fig. 3).

### Major oxides, trace elements, and weathering indices

Both sites are characterized by a Si-rich substrate (Supplementary Table S1) having a considerable content of Al_2_O_3_. The SiO_2_, Al_2_O_3_, MgO, and K_2_O concentrations increase with soil depth (Supplementary Fig. S3; e.g., Al_2_O_3_ valley: p = 0.78, ridge p = 0.77). An inverse trend is seen with CaO and MnO decreasing with depth (CaO valley: p = − 0.38, ridge: p = − 0.65). A general increase in the content of CaO and a less pronounced increase in TiO_2_ are observed from the reference (R1, R2) to the erosional sites (S1, S2, S3, S4) (Supplementary Table S1; e.g., CaO valley: p = 0.70, ridge: p = 0.52). A decrease from hilltop to foot slope is primarily found at both sites for P_2_O_5_ (valley: p = − 0.36, ridge: p = − 0.70) and organic matter (Supplementary Table S1). Overall, the highest concentrations of major oxides were measured at the ridge, except for Na_2_O and SiO_2,_ which were higher in the valley (Supplementary Table S1).

**Figure 4:**
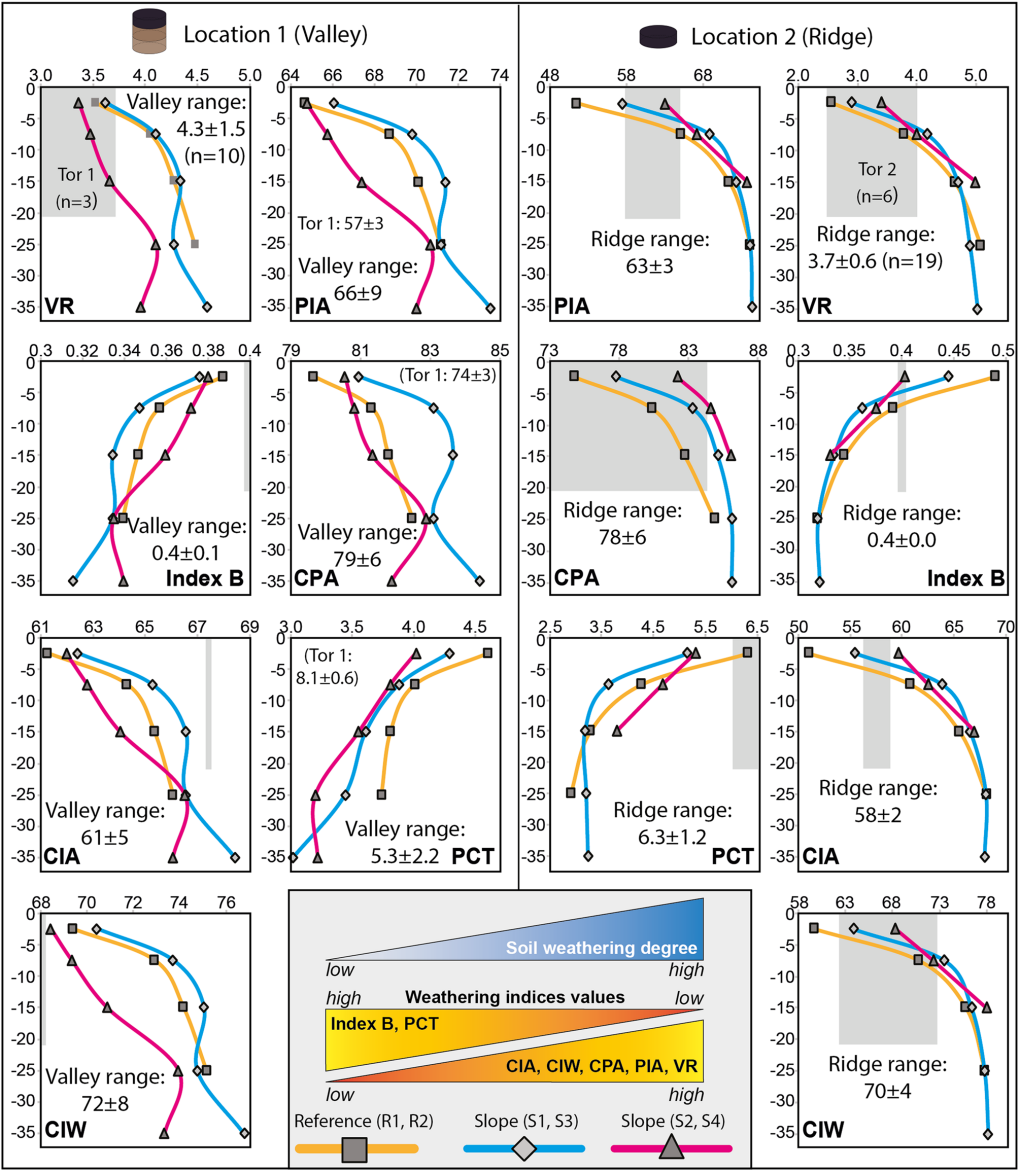
Weathering indices along the soil profiles at reference and slope sites of the two landscape settings (valley, ridge). The y-axis represents the soil depth (in cm) and the x-axis the corresponding average (n = 4) element ratio (see 3.5; Supplementary Table S3). The grey shaded area indicates the range of Tor 1 at the valley (n = 3) and Tor 2 at the ridge (n = 6). In addition, the characteristic range of a larger sample set is provided for the valley (n = 10) and the ridge (n = 19). The coloured triangles in the scheme represent the theoretical relation of soil weathering degree to the various calculated weathering indices: Index B^82^ and PCT^76,86^ decrease while the CIA^83^, CIW^84^, CPA^85^, PIA^40^, and VR^78^ values increase with increasing degree of weathering (see Supplementary Table S4 and “Major and trace element contents and chemical weathering indices” for indices functions).

Nearly all weathering indices (Supplementary Tables S3, S4) show increased weathering intensity with soil depth (e.g., valley correlation for PIA: 0.74, CIA: 0.78, VR: 0.62, CIW: 0.69, Index-B: − 0.78, CPA: 0.58; ridge correlation for PIA: 0.77, CIA: 0.78, VR: 0.81, CIW: 0.77, Index-B: − 0.78, CPA: 0.67) and downhill (e.g., R2 → S3 → S4; Fig. 4). Overall, negative weathering indices with soil depth trends exist at all sites at the ridge and the reference site of the valley. Both sites, S2, and S1, show an irregularity at a depth of about 25 cm. Compared to the adjacent rock tors, the soils at the ridge have a similar weathering intensity in the topsoil, and weathering intensity increases with soil depth. In contrast, in the valley, the majority of weathering indices show that soils are already strongly weathered at all depths, including the surface (Fig. 4).

## Discussion

In undisturbed soils, the δ^13^C ratio becomes typically less negative with decreasing C_org_ contents^25^. Such a correlation can only be found on slope 2 (valley). At all other sites, no correlation exists between the δ^13^C ratio and the C_org_ content. Overall, a correlation between C_org_ and δ^13^C is weak or nonexistent and does not provide the expected (see “Determination of short- and mid-term soil erosion”) evidence of soil stability reported by others^25–27^.

The C_org_ and δ^13^C plots potentially indicate^28^ that all soils might have undergone either a form of soil mixing (R1, R2, S1, S3, S4) or soil redistribution (S2), also evident with a relatively homogeneous δ^13^C depth trend (Supplementary Fig. S2; valley: p = 0.07, ridge: p = 0.01). Yet, the investigated soil profiles have not been ploughed to our knowledge during the last few decades. If this had been the case, the Pu-profiles would look much more homogeneous. Comparing the δ^13^C depth trend with the ^239+240^Pu signal suggests that soil mixing must have occurred before the plutonium fallout or abruptly stopped afterward. Other mechanisms include erosion or vegetation changes. If continuous erosion had been the sole factor, clearer depth trends of δ^13^C with C_org_ likely would have remained^27^. Erosion along slopes may not be a constant process but is accompanied by phases of redeposition and accumulation. In general, the current absence of higher vegetation (e.g., shrubs, trees) in our study area would allow splash erosion^21^ and erosion due to overland flow during occasional storm events to give rise to a less predictable and more uneven depth trend signal of δ^13^C and C_org_. We also must consider that Otago province has a century-long history of vegetation depletion and grazing with the conversion from native vegetation to pasture^13^. Thus, today’s δ^13^C depth trends might just represent the average from different vegetation types, serving as remanents that reflect the change from the native woody vegetation (pre-settlement forest) to modern pasture.

Our field observations, showing wavy to lobate or irregular boundaries between surface and subsurface soil horizons and stony layers occasionally, further indicate some disturbance of the soil profile, likely caused by reworking processes, such as soil creep, colluviation, surface runoff, and possibly past land use changes from tillage (no more active in the last few decades) to pasture (without the formation of terraces). These features are in line with C and N depth patterns, showing occasional inflections (relative maxima or minima) at intermediate depths, and can explain the non-correlation between C_org_ and δ^13^C.

In contrast, the exponential depth functions of ^239+240^Pu in the soil indicate that potential disturbance of the soil by ploughing or redistribution processes is neglibible^29^ and suggest appropriate site selection. The ^240^Pu/^239^Pu atomic ratios at all sites are characteristic of the fallout signature recorded in the southern hemisphere (0.185 ± 0.047; Fig. 3a)^29^. The calculations using MODERN and the PDM gave comparable results. The results of the PDM (or MODERN) and IM conversion models significantly differ at some sites (e.g., S2, S4) and only agree well or moderately at the intermediate slope S1 or S3. The models indicated the lowest soil erosion and accumulation rates at S1. Sites with low inventory change have a high correspondence between the results obtained with MODERN and the PDM^30^.

The erosion rates via IM are unrealistically high, which might be a method-inherent problem. The IM is a relatively simple approach and assumes that the FRN distribution in the soil exhibits an exponential function. At site S4 (Ridge), erosion rates of 2900 [t km^−2^ year^−1^] are calculated, which would correspond to extremely intensively used soils^13^. For example, the mean soil loss rate for European Union erosion-prone land (agricultural, forests, and semi-natural areas) is 246 [t km^−2^ year^−1^]^31^. For permanent cropland uses, average European Union values are only 950 [t km^−2^ year^−1^]. Consequently, the calculated values for site S4 via IM do not seem very probable.

The PDM and MODERN results are further compared to past ^137^Cs investigations. The PDM resulted in annual average erosion rates of 260 [t km^−2^] for the valley and 993 [t km^−2^] for the exposed ridge. Average rates using MODERN are 242 [t km^−2^] for the valley and 1043 [t km^−2^] for the ridge. These values are comparable to the ^137^Cs-results of 420 to 1020 [t km^−2^ year^−1^] for crests and slopes (18° inclination) previously determined for Otago^14^. The foot slope in the valley also has about four times lower soil erosion rates compared to the ridge via PDM (Fig. 3c). With MODERN, even lower accumulation rates were calculated.

From a country-wide perspective, soil redistribution rates in Otago are relatively low compared to other New Zealand regions. For example, on the South Island, the soil erosion rates determined with ^137^Cs were about 4600 [t km^−2^ year^−1^], and deposition rates of 4900 [t km^−2^ year^−1^]^32^ in the Canterbury province (see Fig. 1c) and exceeded all Otago averages in this study (Supplementary Table S0). Surface erosion rates in east Canterbury are in the range of 0–1300 [t km^−2^ year^−1^] with deposition rates of up to 1600 [t km^−2^ year^−1^]^13^. Also, on the North Island of New Zealand, soil erosion rates of 1100–1400 [t km^−2^ year^−1^] are found in the Manawatu area (Fig. 1c). Likewise, the Pukekohe site (Fig. 1c) had mean soil erosion rates of 700–3100 [t km^−2^ year^−1^] out of a total erosion range of 3500–9800 [t km^−2^ year^−1^] and a deposition range of 3400–10,900 [t km^−2^ year^−1^]^13^. The likely reasons for these differences are rainfall and land use. The investigated sites of other authors^13^ were primarily cropland with intensive vegetable production and heavy machinery use. In addition, the North Island experiences greater rainfall (up to 2800 [mm year^−1^]) compared to the semi-arid Otago upland.

Overall, the soil redistribution rates indicate an erosion-dominated regime for our sites. Our study area's environmental conditions (i.e., semi-arid, absence of higher vegetation) suggest that sediments have been removed by aeolian and fluvial processes. The Taieri River (Fig. 1) draining part of East Otago has an annual suspended sediment yield of 56 [t km^−2^] (0.32 Mt annual load, catchment of 5700 km^2^) to the coast^1^ or about 22 [t km^−2^] (Sutton Stream at SH 87^33^). This low river sediment yield is also supported by earlier investigation^2^ where annual sediment yields for Otago Schist and semi-schist areas with low annual rainfall (660 [mm year^−1^]) are in the range of about 22 [t km^−2^] (or 56 [t km^−2^ year^−1^] for 672 [mm year^−1^]). Comparing these river sediment yields with both the ^137^Cs (420–1020 [t km^−2^ year^−1^]) and ^239+240^Pu based rates (260–990 [t km^−2^]) suggests that only about 5–23% has been transported out of the catchment by fluvial processes to the river outlet^2^. This behavior suggests temporal storage of sediments eroded by soil creep and occasional surface runoff (e.g., storm events) along slopes in the river catchment, including the active talweg, before delivery to the sea.

Alternatively, or in addition, we recommend that at our study site, a large amount of about 580–660 [t km^−2^ year^−1^] is either redeposited along slopes or may be attributed to wind erosion, and that soil erosion is wind dominated. In a global context, the Otago (aeolian) erosion rates are in the upper part of measured ranges in areas having similar climatic conditions. Wind erosion in uncultivated grassland of the Mongolian plateau (annual precipitation 132–353 mm at 1000–2000 masl) was estimated using ^137^Cs and lay in a range of 65–170 [t km^−2^ year^−1^]^34^. In cultivated areas, the maxima were near 420 [t km^−2^ year^−1^]^35^. Furthermore, two-year observations in the semi-arid Chinese Loess Plateau (annual precipitation 423 mm) resulted in average wind erosion rates of 588 [t km^−2^ year^−1^] using ^7^Be^77^. The Otago upland landscape also provides additional evidence to support continuous wind erosion—deeply carved tafone (see Fig. 1f,g). Most of the local schist tors (large residual rocks exposed at the ground surface after erosion and still rooted in bedrock) exhibit typical tafone (prominent, cavernous weathering features), which are common in semi-arid regions^36,37^. Tafone formation can have a polygenetic origin because of a complex mix of weathering mechanisms, such as chemical weathering, temperature variations, salt weathering, and wind abrasion. We consider wind erosion^9^ to be the driving force for shaping the Otago tors. A recurring stream of particles is needed to reshape the rock outcrops to become abraded. An annual wind erosion ~ 580–660 [t km^−2^] of particles can certainly perform such reshaping. The most robust abrasion features were seen on northwest-oriented tors^38^, consistent with the dominant mean wind direction (see “Sampling strategy and study area”).

While these observations are consistent with substantial wind erosion, additional systematic studies are needed to quantify it. If wind erosion is a dominant factor at Otago, then landscape position is critical with higher erosion at exposed ridges, relative to valley sites. Thus, in future studies, one should consider that ridge crests are potentially less suitable for understanding the long-term surface exposure history of the area^39^.

Yet, intact exponential Pu activity depth trends in our samples (Fig. 3b) demonstrate that the FRNs have remained largely unaffected by the afore-mentioned redistribution processes, at least during the last ~ 60 years, with soil formation likely overcoming (and overprinting) morphodynamic processes. However, traces of long-term differences controlled by slope dynamics and landscape position could be reflected in element content depth patterns in the soils.

The leaching intensity of chemical weathering reactions is governed by the minerals in the rock, the mineral residence time, temperature, pH, redox potential, the solubility of minerals, and foremost by the abundance and movement of water^40^. When climate (temperature and precipitation), vegetation cover, and land use, soil properties (bulk density, pH, etc.), and the underlying bedrock material are mostly uniform, then topography and landscape position become the controlling factors over insolation, wind exposure, and hydrological flow patterns^41^. These determine the leaching processes, including the wash-out of weathering products and the chemical weathering rate and intensities.

At both ridge and valley sites, the concentrations of the mobile cations K, Ca, Mg, Sr, and Ba, as well as less soluble cations (e.g., Fe, Al, Si, Zr, Nb), increase downslope (e.g., p = 0.69 for Ba along ridge slope; p = 0.74 for Zr along valley slope; Supplementary Tables S1, S2; Supplementary Figs. S3, S4).

This increasing downslope pattern and heterogeneity of mobile cations between the sampling sites series (R1-S1-S2; R2-S3-S4) is differently accentuated for different elements between the study location (Supplementary Fig. S3; e.g., CaO valley: p = 0.70, ridge: p = 0.52; P_2_O_5_ valley: p = − 0.36, ridge: p = − 0.70; MgO valley: p = 0.19, ridge: p = 0.67). Also, less soluble cations show this downhill difference (e.g., Zr valley: p = 0.74, ridge: p = 0.11) and even reversals (e.g., Rb valley: p = − 0.89, ridge p = 0.78; Ba valley: p = − 0.59, ridge: p = 0.69). This could be partly related to subsurface and Hortonian overland flow^42,43^. Soluble cations dissolved in infiltrating water could be transported laterally along the slope dip through the soil pore system. Relatively insoluble ions, especially Fe^3+^ Al^3+^ and Si^4+^, could be transported downhill by physical erosion–deposition processes. Ions Si^4+^, Zr^4+^, Fe^3+^, and Al^3+^ could still be largely included in the coarse fraction, i.e., in the primary minerals (quartz, zircon, micas and feldspars) from the Otago Schist rock and to a lesser extent in neoformed clays or oxyhydroxides and transported downhill by physical erosion–deposition processes.

Otago has been farmed since the 1800s, accompanied by phosphate-based fertilizer application since the late 1800s, affecting local soil chemistry^44,45^. In particular, increases in near-surface soils of MnO, P_2_O_5_ (Supplementary Table S1), or sulphur concentrations could be attributed to phosphate-bearing fertilizer addition^44^. The P-based fertilizer application also can cause an enrichment of specific heavy metals (Cd, Cu, Zn, Ni, Hg, As, Cr, Pb)^45^ in soils (Supplementary Table S2). Comparing the major element contents of the parent material (Supplementary Table S6) and soils (Supplementary Table S1) further reflects the enrichment, which, however, can also have been contributed by the local schist (e.g., P_2_O_5_ of schist: valley-soils is F: 3.22 to F_α_: 2.06 and schist: ridge-soils is F: 2.72 to F_α_: 1.79). The schist varies from 900 to 5700 ppm P_2_O_5_ (average ~ 2600 ppm), while soils range from about 140 to 9000 ppm P_2_O_5_ (average ~ 3000 ppm). Also, an increase of calcium in the topsoil (~ 20 cm) is often associated with forest and fertilization. However, calcium enrichment due to fertilization (e.g., Ca(H_2_PO_4_)_2_) is not supported by XRF measurements nor by the heterogeneity of variance among the local schist and soils (F-test CaO of schist: valley-soils is F: 8.62 to F_α_: 2.06 and schist: ridge-soils is F: 2.21 to F_α_: 1.84). The maxima and total averages among the bedrock and the soil, the schist ranges from 1200 to 19,500 ppm CaO (average 12,200 ppm), and in the soil from about 7500 to 18,500 ppm CaO (average ~ 12,000 ppm). However, the high CaO concentration at the topsoil may also be explained by plant uplift and could indicate prior forestation^46^. We tend to consider the decline of CaO with soil depth (Supplementary Fig. S3; valley: p = − 0.38; ridge: p = − 0.65) because of surface enrichment by schist tor weathering. The systematic CaO surface increase downhill (Supplementary Fig. S3) at both sites (ridge and valley) suggests a natural mechanism, e.g., gravitational rock material accumulation. However, a contribution due to fertilization or prior forestation cannot be entirely ruled out.

The selected weathering indices show increasing weathering degree depth trends across all sights (Fig. 4; “Major oxides, trace elements, and weathering indices”). This is unusual as the highest weathering degree would be expected at the topsoils since the soil subsurface should experience a recurring rejuvenation of the weathering front of the bedrock below. In general, the weathering indices indicate incipient to moderate chemical alteration^47^. This pattern corresponds to relatively young soils or soils having a fast turnover rate due to erosion and, thus, a low residence time for soil particles^48^. With its high soil erosion rates, the exposed ridge (Fig. 3c) appears to have a rapid physical removal of primary minerals, resulting in the most expected weathering patterns (Fig. 4) due to the depletion of fresh material. The depletion or enrichment of major elements downhill at both sites further supports the physical removal (Supplementary Table S1, Supplementary Fig. S3). For example, at the valley, we see an increase in the first 5 cm of the indices (Supplementary Table S4) from R1 to S1 by 24.6%, and from R2 to S2 by about 57.2%, K_2_O increased by about 2.6%, and 16.8% respectively (see above). But since the downhill increase (respectively R1-S1 and R1-S2) of, e.g., MgO (9.1% and 29.2%;), CaO (4.2% and 15.4%) or Na_2_O (5.3% and 0.7%) is lower compared to Al_2_O_3_, we still observe an increase of soil weathering downhill (see equations in Supplementary Table S4; Fig. 4; F-test valley for PIA and CIA of R1: S2 are F: 1.10 to F_α_: 2.23 and F: 1.07 to F_α_: 2.23; ridge R2: S3 are F: 1.90 to F_α_: 2.31 and F: 1.88 to F_α_: 2.31, respectively).

Overall, the valley has more irregular depth trends of weathering indices, with a recurrent flexure below ca. 15 cm depth, which is consistent with C and N depth patterns. These findings seem related to downslope soil redistribution processes and are also found in trace element distributions (Supplementary Fig. S4; see above). The consistency of the pattern suggests that such geochemical data and associated weathering indices can be reliable and indicative of the local geomorphic dynamics, despite some caution being compulsory because an anthropogenic input potentially sourced from (past) fertilization practice cannot be entirely ruled out.

The valley soils' weathering degree is distinctly higher than the ride (Fig. 4; F-test valley: ridge PIA are F: 0.15 to F_α_: 0.63 and CIA are F: 0.16 to F_α_: 0.63). For example, comparing the lowest weathering values among indices (see Supplementary Tables S3 and S4) at the two locations, the PIA resulted in a ~ 26% higher (R1: 64.7, R2: 51.4), and the CIA resulted in a ~ 20% higher (R1: 61.3, R2: 51.0) weathering intensity in the valley than at the ridge (Supplementary Table S3). Thus, a tripling (IM: 996 vs. 2922 [t km^−2^ year^−1^]) or quintupling (PDM: 260 vs. 1356 [t km^−2^ year^−1^]) of the soil erosion rates (valley vs. ridge average) resulted in a 20–26% lower degree of soil weathering at the ridge compared to the valley, due to the shorter dwell time of minerals and soil particles. This observation is in accordance with the general understanding that the residence time of minerals within soils is sensitive to physical denudation rates in geomorphically dynamic landscapes^49–51^.

However, the role of tors in the context of providing fresh mineral materials is still in question. In regions having a dry climate with substantial diurnal changes in temperature can cause mechanical or physical disintegration of rocks or abrasion of rock surfaces^52^, which is the case in Otago^53^. The weathering indices show that the reference sites closest to the bedrock outcrops have the lowest soil weathering degree. Thus, it appears that a soil rejuvenation process is occurring at the hilltop. The rock outcrops (tors) at each hilltop (Fig. 1d,e) could serve as a rejuvenation source as physical and chemical weathering can cause a regular or abrupt input of fresh mineral material derived from tor degradation^53^. We imagine that at Otago, the tor-derived mineral supply must be greater than the contribution of the bedrock weathering front at the soil–bedrock boundary to explain the inverse weathering degree depth trends (Fig. 4). Together with the lack of higher vegetation and with, e.g., splash erosion, occasional overland flow, or wind erosion a dispersion of the degraded material downslope would cause soils within the transport range of the tor-derived material to also have an inversion in their weathering degree depth trends—matching our data.

## Conclusions

In the dry oceanic climate regime of East Otago, average soil erosion rates from fallout radionuclides indicate lower erosion in the valley (260 [t km^−2^ year^−1^]) and (~ four times) higher erosion on the ridge (990 [t km^−2^ year^−1^]). The soil erosion rates measured using^239+240^Pu are consistent with ^137^Cs-based rates of previous investigations. The Pu-inventory method generally gave the highest values—in terms of erosion and accumulation—but these values seem unrealistic due to methodological deficiency.

The weathering indices indicate weaker chemical mineral decomposition of up to 26% at the ridge compared to the valley floor, supporting faster soil erosion and shorter soil particle residence times at the ridge. The soil weathering degree increases with distance to tors (large residual rocks) and is inverted at all sites, as the soil weathering decreases with soil depth. There appears to be a higher fresh mineral supply from the soil surface than from the underlying bedrock weathering front. We hypothesize that residual rock tors served as the predominant input source of fresh mineral material to cause an increasing weathering degree gradient downhill and with soil depth.

Comparing these soil erosion rates to river sediment yields suggests that no more than 23% of the sediment has been transported out of the catchment by fluvial processes to the river outlet. Abraded and hollowed rock tors of East Otago would also support the hypothesis that wind erosion may account for a substantial portion of the missing sediment flux.

## Material and methods

### Sampling strategy and study area

The investigation sites for this study were in the east of the Otago upland (Fig. 1a,b; Table 1). The East Otago upland can be hilly (12°–25° slope angle; Fig. 1d,e), and the minimum elevation is frequently 300 masl. Two hillslopes nearby (~ 5 km; Fig. 1d,e) having differing landscape settings (Fig. 1b) were included in our sampling strategy, one in a valley (location 1; Fig. 1d) and the other on an exposed ridge (location 2; Fig. 1e; Table 1). At each location, one reference (‘R’, with 4 replicates) and two soil pits (each having 4 replicates) along the slopes (‘S’) were sampled with a soil corer (100 cm^3^). The investigation sites at the foot of each hill were near gullies (vegetated channels), but this is unavoidable in the frequently gullied Otago landscape. Land use is for sheep farming, but land disturbance by ruminants was not observed, and the land has not been ploughed. At each reference and investigation plot, two pits with two sampling series were taken, resulting in four replicates per soil depth and sampling spot.

The basement rocks in the study area are Rakaia Terrane, composed mainly of Permian- to Jurassic sandstones and mudstones now metamorphosed to schist rock (greenschist facies) and tectonically deformed^54,55^. The schist can form prominent tors of isolated rock in the study area^56^ that are commonly abraded and caved (Fig. 1f,g). The typical north-westerly winds are often dominated by Foehn from the New Zealand Southern Alps mountain range^57^. The Otago area around Middlemarch township experiences about 55 days of wind gusts > 61 [km h^−1^] and regular maximums of about 130 [km h^−1^]^58^. Our investigated sites' apparent wind direction is primarily perpendicular to the northeast-southwest ridge axes. Such aeolian forces in East Otago significantly influence shaping landscapes with reduced near-surface soil water content^31^.

The Otago climate suggests moderate pluvial erosion, negligible mass movement, high wind erosion (Fig. 1c) and a dominance of (slight–moderate) chemical weathering^59^. Otago is one of the driest, hottest, but also coldest areas in New Zealand^60^. The Southern Alps cause a rain shadow over parts of Otago (Fig. 1b), resulting in a semi-arid continental climate^61^. Precipitation reaches lows of about 300–350 [mm year^−1^] on the valley floors, and highs of about 2250 [mm year^−1^] on the highest peaks (1450 masl)^62^. Most area is classified as a temperate oceanic climate after the Köppen–Geiger climate classification^63^. The average temperature is about 15.2 °C; the average annual temperature has increased continuously over the last 150 years^64^. The study area has never been glaciated^65^.

### Physical, chemical, organic C, and stable carbon isotopes analyses

All 102 soil samples were oven dried (80 °C) for 48 h and sieved to < 2 mm (fine earth). This enabled the determination of the proportion of rock fragments (soil skeleton). The bulk soil density was obtained from the dry weight of the 100 cm^3^ soil cores before fine milling (< 50 µm). Soil pH (in 0.01 M CaCl_2_) was measured on air-dried fine earth samples and a soil:solution ratio of 1:2.5. Loss on ignition (LOI) was determined once by dry-ashing for Pu-analysis (16 h, 550 °C) and secondly by igniting 2 g of oven-dried fine earth at 1000 °C for 2 h. The major and trace element content of oven-dried fine earth (1000 °C) was measured using X-ray fluorescence (XRF)^66^. About 5 g of fine earth was milled to < 50 µm and analyzed as loose powder in sample cups using an energy dispersive XRF spectrometer (SPECTRO X-LAB 2000, SPECTRO Analytical Instruments, Germany). The organic carbon (C_org_) contents were obtained by measuring 0.1 g of finely milled soil material in tin capsules with a Leco^®^ C–H–N elemental analyzer (Leco TruSpec Micro Analyser). The EDTA standard (CAS Nr.: 20824-56-0) and the Soil-Leco (Part. No.: 502-308) were used for standardization. The δ^13^C isotopic ratios were measured with a Picarro analyzer for isotopic CO_2_ (Combustion Module-Cavity Ring Down Spectroscopy (CM-CRDS), Sunnyvale, California, USA). Instrumental precision is < 0.1‰. Therefore, soil powder of the milled fine earth was weighed (~ 0.1 g) into tin capsules and combusted at 950 °C. The released CO_2_ was measured with a CRDS analyzer (G2131-i). An internal standard (30B00GW9 Chernozem 2013) was used for every six samples to correct for potential drift of the Picarro analyzer (< 0.5‰) in the C and δ^13^C values.

### Determination of short- and mid-term soil erosion

We used ^239+240^Pu to assess soil redistribution rates of the recent past (last about six decades). These fallout radionuclides provide an average redistribution rate for the time since emission during nuclear weapons tests (maximum in 1963–1964^67^). Average soil redistribution rates are calculated based on the differences in Pu-activity [Bq m^−2^] among inclined investigation sites (e.g., slopes) and a flat reference site. It is assumed that ‘accumulation’ sites (e.g., valleys, slope foot) have a higher, while ‘erosion’ sites (e.g., ridges, steep slopes) have a lower, Pu-activity than their local ‘reference’ site.

The inventory of the ^239+240^Pu activities [Bq m^−2^] serves as a basis for the calculation of soil redistribution rates (erosion/accumulation):

1 $$I=\frac{1}{a}{\sum }_{i}{M}_{i}{C}_{i},$$

where *a* = horizontal cross-sectional area (m^2^), *M*_*i*_ = mass (kg) of the *i-*th sample depth increment and *C*_*i*_ = activity (Bq kg^−1^) of the *i-*th sub-sample depth increment.

Redistribution rates of soils were then obtained by comparing the isotope inventory for an investigation point (potential erosion or accumulation site) with a stable local reference inventory where neither soil erosion nor soil accumulation is expected. The current FRN methods to calculate erosion rates are often at their limit with stony soils, arid and semi-arid areas with scarce vegetation cover, and mountain regions in general^68^. For comparison, we used three different models to convert ^239+240^Pu inventories into soil redistribution rates:

1. The profile distribution model (PDM) for uncultivated soils^23,24^ uses a simple numerical (exponential) function to represent the vertical distribution of the measured Pu:

2 $${I}^{\prime}\left(x\right)={I}_{ref}\left(1-{e}^{\frac{{M}_{x}}{{h}_{0}}}\right),$$

where *I′*(*x*) is the amount of the isotope inventory (Bq m^−2^) above the depth (x), *I*_*ref*_ is the reference inventory (Bq m^−2^) (location 1), *M*_*x*_ is the fine earth mass (kg m^−2^) between top and actual depth (x), *h*_0_ is the profile shape factor (kg m^−2^). The *E*_*soil*_ is the soil erosion rate (t km^−2^ year^−1^) was calculated using

3 $${E}_{soil}=\frac{10}{t{-t}_{0}}\times ln\left(1-\frac{{I}_{ref}{-I}_{inv}}{{I}_{ref}}\right)\times {h}_{0}\times 100,$$

where t is the year of sampling (2017), t_0_ is the 1963 (maximum peak of thermonuclear weapon testing) and I_inv_ is the investigation site inventory (Bq m^−2^).

2. The inventory method (IM)^69^ calculates the loss of soil, L [t km^−2^ year^−1^], also for uncultivated and unploughed soils:
  4 $$L=\frac{1}{\alpha P}\times ln\left(1-\frac{{I}_{ref}{-I}_{inv}}{{I}_{ref}}\right)\times \rho \times \frac{{10}^{4}}{t{-t}_{0}},$$
  where *α* is the coefficient of the least square exponential fit of the profile depth to activity^70^, *P* is the particle size correction factor, and ρ is the represents the average fine earth density [g cm^−3^]. For the IM models, *P* values of 1, 1.2, and 1.5 were used^69^.

3. MODERN (Modelling Deposition and Erosion rates with RadioNuclides^71,72^): The underlying idea behind the model is the comparison of the depth profile of the reference site with the total inventory of a sampling site. MODERN models the depth profile of FRN of the reference site as a step function with a specific increment. It does not make any assumptions or generalisations about the shape of the reference site. Additionally, soil layers can be added above and below the measured layers to better estimate erosion or deposition^23,24^.

A further hint about soil redistribution, but more qualitatively than quantitatively, gives the C_org_ and δ^13^C and the correlation between these two parameters. Generally, a decrease in the total soil C_org_ and an enrichment of heavier stable carbon isotope (δ^13^C) with increasing soil depth can be expected due to natural fractionation during organic matter decomposition^73^. This negative linear correlation between δ^13^C and C_org_ mid-term disturbances (few millennia^25^) and soil stabilities (e.g., presence of bioturbation^27^) in soils can be qualitatively assessed.

### Sample treatment and ^239+240^Pu measurement

All samples were dry-ashed (16 h at 550 °C) and spiked with ~ 0.005 Bq of a ^242^Pu tracer (Harwell, 0.00253 Bq or 17.07 pg to each sample). Plutonium was leached out in nitric acid (16 M at 80 °C) overnight and subsequently was isolated from the solution using Pu-selective TEVA resin (EIChrom, Lisle IL^69^). The samples were analysed for ^239+240^Pu isotopes at the Universidad de Cádiz using a Thermo X7 quadrupole ICP-MS (Inductively coupled plasma mass spectrometry) instrument equipped with a high-efficiency ultrasonic nebulizer system (CETAC U-5000AT). The isotopic masses of 235, 238, 239, 240, and 242 were acquired. A detection limit of 0.002 [Bq kg^−1^] of ^239+240^Pu was obtained for samples of ~ 7 g of dry-ashed material with a measurement error of < 3% for ^239+240^Pu activities > 1 [Bq kg^−1^]. The individual mass ratios were corrected by taking into account the mass bias factor and the UH + /H + ratio, to determine the ^240^Pu/^239^Pu atom ratio and ^239+240^Pu activity. Data quality was evaluated by analyzing blanks (soils and rocks devoid of Pu), duplicates and control samples having known ^239+240^Pu activity. Additional re-measurement of ^239+240^Pu concentration (relative to a ^242^Pu spike) of selected samples were performed at the University of Zurich using an Agilent 8800 triple quadrupole ICP-MS equipped with an ESI Apex-IR nebulizer. The resulting ^239+240^Pu activity was normalized to the Universidad de Cádiz results using linear regression.

### Major and trace element contents and chemical weathering indices

Chemical weathering transforms primary minerals in the parent rock to neogenic minerals (e.g., clay minerals, iron oxyhydroxides) to generate soil, while soluble elements are leached out. In general, mineral solubility is based on its elements' ionic potential (charge:radius). Ions with low ionic potential (e.g., Na, K, Ca, Rb, Sr, Ba, La, Mg, Li, Cs, etc.) are more soluble than ions with high ionic potential (e.g., Ti, Zr, Nb, Al, Be, Sc, Ce, Th, etc.)^74^. As a result, a natural depletion of mobile and enrichment of immobile major and trace elements (“Physical, chemical, organic C, and stable carbon isotopes analyses”) in soil takes place compared to the underlying bedrock. These characteristic alterations of minerals gave rise to various weathering indices (see Supplementary Table S4) that characterise the degree of weathering. In undisturbed soils, the weathering degree decreases with increasing soil depth and increases with soil surface age^75,76^. We tested several geochemical weathering proxies as potential tracers of soil mobility (Supplementary Table S4).

## Supplementary Information

Supplementary Figure S1.

Supplementary Figure S2.

Supplementary Figure S3.

Supplementary Figure S4.

Supplementary Table S0.

Supplementary Table S1.

Supplementary Table S2.

Supplementary Table S3.

Supplementary Table S4.

Supplementary Table S5.

Supplementary Table S6.

## Data Availability

All data is available either in the main body of the manuscript or in the supplementary files. In addition, data will be freely available on www.geraldraab.com or by e-mail request.
