# Supplementary material for: Soil weathering dynamics and erosion in a dry oceanic area of the southern hemisphere (Otago, New Zealand)
Source: Sci Rep. 2022 Nov 17;12:19803. doi: 10.1038/s41598-022-23731-7 (PMC9672066; doi:10.1038/s41598-022-23731-7)
Supplement: Supplementary file 5 — Supplementary Table S0. [file 41598_2022_23731_MOESM5_ESM.doc]

**Table S0**: Calculated soil erosion rates of ^239+240^Pu investigated soil pits of Location 1 (S1,S2) and Location 2 (S3,S4) using the inventory method (IM) after Lal et al. (2013), the profile distribution model (PDM) after Walling & He (1999) and Zhang et al. (1990). Various particle size correction factors (PM) have been applied. Soil redistribution rates were individually calculated with each reference site. Figure 2 provides a more graphical illustration of the data.

|  | **Inventory method (IM)** | | |  | **PDM** |  |  |
| --- | --- | --- | --- | --- | --- | --- | --- |
|  | PM = 1 | PM = 1.2 | PM = 1.5 |  |  |  |  |
|  | [t km^-2^ yr^-1^] | [t km^-2^ yr^-1^] | [t km^-2^ yr^-1^] |  | [t km^-2^ yr^-1^] |  |  |
| **Location 1 (Valley) – Reference 1** | | | | | | | |
| Slope 1 (S1) |  |  |  |  |  |  |  |
| L1-S1-P1-1 | -423 | -352 | -282 |  | -114 |  |  |
| L1-S1-P1-2 | -3,186 | -2,655 | -2,124 |  | -910 |  |  |
| L1-S1-P2-1 | -49 | -41 | -33 |  | -14 |  |  |
| L1-S1-P2-2 | 2,296 | 1,913 | 1,531 |  | 693 |  |  |
| **Average** | **-340** | **-284** | **-227** |  | **-86** |  |  |
| **Std.error** | **65.7** | **60.0** | **53.6** |  | **35.5** |  |  |
|  |  |  |  |  |  |  |  |
| Slope 2 (S2) |  |  |  |  |  |  |  |
| L1-S2-P1-1 | -1,105 | -921 | -737 |  | -273 |  |  |
| L1-S2-P1-2 | -2,084 | -1,737 | -1,389 |  | -540 |  |  |
| L1-S2-P2-1 | -1,719 | -1,432 | -1,146 |  | -465 |  |  |
| L1-S2-P2-2 | -1,699 | -1,416 | -1,133 |  | -460 |  |  |
| **Average** | **-1,652** | **-1,376** | **-1,101** |  | **-435** |  |  |
| **Std.error** | **27.9** | **25.5** | **22.8** |  | **14.8** |  |  |
|  |  |  |  |  |  |  |  |
| Total Average | **-996** | **-830** | **-664** |  | **-260** |  |  |
| Std. error | **46.8** | **42.7** | **38.2** |  | **25.1** |  |  |
|  |  |  |  |  |  |  |  |
| **Location 2 (Ridge) – Reference 2** | | | | | | | |
| Slope 3 (S3) |  |  |  |  |  |  |  |
| L2-S1-P1-1 | -1'847 | -1'539 | -1'231 |  | -923 |  |  |
| L2-S1-P1-2 | -676 | -563 | -450 |  | -343 |  |  |
| L2-S1-P2-1 | -1'208 | -1'006 | -805 |  | -642 |  |  |
| L2-S1-P2-2 | -1'099 | -916 | -733 |  | -611 |  |  |
| Average | **-1'207** | **-1'006** | **-805** |  | **-630** |  |  |
| Std.error | **28.4** | **27.8** | **24.9** |  | **21.3** |  |  |
|  |  |  |  |  |  |  |  |
| Slope 4 (S4) |  |  |  |  |  |  |  |
| L2-S2-P1-1 | -1'105 | -921 | -736 |  | -647 |  |  |
| L2-S2-P1-2 | -3'554 | -2'961 | -2'369 |  | -1'728 |  |  |
| L2-S2-P2-1 | -3'063 | -2'553 | -2'042 |  | -1'356 |  |  |
| L2-S2-P2-2 | -3'965 | -3'304 | -2'643 |  | -1'695 |  |  |
| Average | **-2'922** | **-2'435** | **-1'948** |  | **-1'356** |  |  |
| Std.error | **49.3** | **45.0** | **40.3** |  | **31.0** |  |  |
|  |  |  |  |  |  |  |  |
| Total Average | **-2'064** | **-1'720** | **-1'376** |  | **-993** |  |  |
| Std. error | **38.8** | **36.4** | **32.6** |  | **26.2** |  |  |
|  |  |  |  |  |  |  |  |

Gerald Raab^a,b*^, Markus Egli^a^, Kevin P. Norton^c^, Adam P. Martin^d^, Michael E. Ketterer^e^, Dmitry Tikhomirov^a^, Rahel Wanner^f^, Fabio Scarciglia^g^

^a^ Department of Geography, University of Zurich, Winterthurerstrasse 190, 8057 Zurich, Switzerland

^b^ Department of Earth and Environmental Sciences, Dalhousie University, PO BOX 15000, 1459 Oxford Street, Halifax

^c^ School of Geography, Environment and Earth Sciences, Te Herenga Waka, Victoria University of Wellington, PO Box 600, 6140 Wellington, New Zealand

^d^ GNS Science, Private Bag 1930, Dunedin, New Zealand

^e^ Chemistry and Biochemistry, Northern Arizona University, Box 5698, Flagstaff, AZ 86011-5698, USA

^f^ Institute of Natural Resource Sciences, Zurich University of Applied Sciences, Grüental, 8820 Wädenswil, Switzerland

^g^ Department of Biology, Ecology and Earth Sciences (DiBEST), University of Calabria, Via P. Bucci – Cubo 15B, 87036 Arcavacata di Rende (CS), Italy

*Corresponding author. Tel.: +41 44 635 65 27; Fax: +41 44 6356848.

E-mail address: gr.science@gmx.at (G. Raab).
