# Supplementary material for: Soil weathering dynamics and erosion in a dry oceanic area of the southern hemisphere (Otago, New Zealand)
Source: Sci Rep. 2022 Nov 17;12:19803. doi: 10.1038/s41598-022-23731-7 (PMC9672066; doi:10.1038/s41598-022-23731-7)
Supplement: Supplementary file 6 — Supplementary Table S1. [file 41598_2022_23731_MOESM6_ESM.doc]

**Table S1**: Total elemental content of soil (given in oxides), of all soil samples LOI = loss on ignition; OM = organic matter, whereby the total organic C was determined via CHN-analyzer and multiplied by the factor 1.72; IVC = Inorganic volatile compounds estimated by LOI minus OM.

|  | Depth | Na_2_O | | MgO | | AL_2_O_3_ | | SiO_2_ | | P_2_O_5_ | | K_2_O | | CaO | | TiO_2_ | | MnO | | Fe_2_O_3_ | | LOI | | OM | | IVC | | Sum | |
| --- | --- | --- | --- | --- | --- | --- | --- | --- | --- | --- | --- | --- | --- | --- | --- | --- | --- | --- | --- | --- | --- | --- | --- | --- | --- | --- | --- | --- | --- |
|  | [cm] | [g kg^-1^] | | [g kg^-1^] | | [g kg^-1^] | | [g kg^-1^] | | [g kg^-1^] | | [g kg^-1^] | | [g kg^-1^] | | [g kg^-1^] | | [g kg^-1^] | | [g kg^-1^] | | [g kg^-1^] | | [g kg^-1^] | | [g kg^-1^] | | [g kg^-1^] | |
| **Location 1 (Valley)** | |  | |  | |  | |  | |  | |  | |  | |  | |  | |  | |  | |  | |  | |  | |
| **Reference Site** |  |  | |  | |  | |  | |  | |  | |  | |  | |  | |  | |  | |  | |  | |  | |
| **L1-R1-P1-1** | 0-5 | 17.7 | | 8.6 | | 117 | | 584 | | 2.9 | | 20 | | 11.0 | | 6.0 | | 0.5 | | 33 | | 199 | | 141 | | 58 | | 1000 | |
|  | 5-10 | 16.9 | | 9.5 | | 133 | | 633 | | 2.4 | | 23 | | 10.7 | | 7.0 | | 0.4 | | 37 | | 128 | | 80 | | 48 | | 1000 | |
|  | 10-20 | 19.5 | | 9.7 | | 139 | | 670 | | 1.7 | | 21 | | 9.3 | | 6.8 | | 0.4 | | 36 | | 86 | | 50 | | 37 | | 1000 | |
|  | 20–25 | 19.6 | | 9.5 | | 141 | | 678 | | 1.6 | | 22 | | 9.0 | | 7.2 | | 0.4 | | 37 | | 75 | | 38 | | 37 | | 1000 | |
| **L1-R1-P1-2** | 0-5 | 18.3 | | 8.9 | | 114 | | 576 | | 3.1 | | 20 | | 11.8 | | 6.0 | | 0.6 | | 32 | | 209 | | 151 | | 58 | | 1000 | |
|  | 5-10 | 18.3 | | 10.1 | | 134 | | 627 | | 2.5 | | 22 | | 10.6 | | 6.7 | | 0.4 | | 35 | | 134 | | 87 | | 48 | | 1000 | |
|  | 10-20 | 17.2 | | 9.6 | | 138 | | 654 | | 2.1 | | 23 | | 10.1 | | 6.9 | | 0.4 | | 37 | | 103 | | 61 | | 42 | | 1000 | |
|  | 20–25 | 18.2 | | 9 | | 142 | | 676 | | 1.5 | | 22 | | 8.9 | | 7.0 | | 0.4 | | 37 | | 77 | | 39 | | 38 | | 1000 | |
| **L1-R1-P2-1** | 0-5 | 16.7 | | 8 | | 107 | | 567 | | 3.7 | | 19 | | 11.1 | | 5.8 | | 0.5 | | 30 | | 232 | | 169 | | 63 | | 1000 | |
|  | 5-10 | 18.4 | | 7.9 | | 120 | | 677 | | 2.1 | | 20 | | 8.6 | | 6.3 | | 0.3 | | 32 | | 107 | | 68 | | 39 | | 1000 | |
|  | 10-20 | 17.1 | | 8.7 | | 129 | | 688 | | 1.7 | | 23 | | 8.3 | | 7.2 | | 0.3 | | 36 | | 82 | | 44 | | 39 | | 1000 | |
|  | 20–25 | 16.0 | | 9.3 | | 138 | | 721 | | 1.4 | | 24 | | 8.8 | | 7.4 | | 0.3 | | 37 | | 37 | | 31 | | 5 | | 1000 | |
| **L1-R1-P2-2** | 0-5 | 15.9 | | 8.4 | | 105 | | 553 | | 3.6 | | 19 | | 11.4 | | 5.7 | | 0.5 | | 29 | | 249 | | 183 | | 66 | | 1000 | |
|  | 5-10 | 17.0 | | 8.4 | | 119 | | 661 | | 2.3 | | 21 | | 9.4 | | 6.6 | | 0.3 | | 33 | | 121 | | 77 | | 44 | | 1000 | |
|  | 10-20 | 18.2 | | 8.7 | | 127 | | 686 | | 1.9 | | 23 | | 9.2 | | 6.9 | | 0.4 | | 35 | | 84 | | 46 | | 39 | | 1000 | |
|  | 20–25 | 17.7 | | 9.3 | | 133 | | 684 | | 2.0 | | 25 | | 9.3 | | 7.2 | | 0.4 | | 38 | | 75 | | 38 | | 38 | | 1000 | |
| **Slope 1** |  |  | |  | |  | |  | |  | |  | |  | |  | |  | |  | |  | |  | |  | |  | |
| **L1-S1-P1-1** | 0-5 | 18.8 | | 10.5 | | 134 | | 571 | | 2.7 | | 21 | | 12.0 | | 6.8 | | 0.4 | | 38 | | 184 | | 130 | | 54 | | 1000 | |
|  | 5-10 | 18.2 | | 10.9 | | 147 | | 608 | | 2.2 | | 24 | | 11.6 | | 8.0 | | 0.4 | | 43 | | 127 | | 79 | | 48 | | 1000 | |
|  | 10-20 | 17.9 | | 11.2 | | 151 | | 622 | | 1.8 | | 24 | | 11.3 | | 8.3 | | 0.4 | | 45 | | 108 | | 60 | | 48 | | 1000 | |
|  | 20-30 | 17.8 | | 11.4 | | 159 | | 626 | | 1.7 | | 25 | | 11.1 | | 9.1 | | 0.4 | | 48 | | 91 | | 44 | | 47 | | 1000 | |
|  | 30-40 | 18.7 | | 12.5 | | 169 | | 634 | | 1.3 | | 24 | | 10.9 | | 10.4 | | 0.4 | | 52 | | 67 | | 21 | | 46 | | 1000 | |
| **L1-S1-P1-2** | 0-5 | 17.1 | | 10 | | 127 | | 546 | | 3.0 | | 23 | | 12.3 | | 7.1 | | 0.4 | | 40 | | 214 | | 144 | | 70 | | 1000 | |
|  | 5-10 | 17.1 | | 10.7 | | 146 | | 597 | | 2.3 | | 26 | | 11.6 | | 8.1 | | 0.4 | | 45 | | 137 | | 82 | | 55 | | 1000 | |
|  | 10-20 | 16.3 | | 10.8 | | 157 | | 634 | | 1.8 | | 25 | | 10.6 | | 8.5 | | 0.4 | | 46 | | 89 | | 44 | | 45 | | 1000 | |
|  | 20-30 | 19.4 | | 11.4 | | 164 | | 639 | | 1.6 | | 23 | | 10.5 | | 8.8 | | 0.4 | | 46 | | 76 | | 30 | | 46 | | 1000 | |
|  | 30-40 | 17.9 | | 12.3 | | 171 | | 632 | | 1.5 | | 25 | | 10.7 | | 10.5 | | 0.4 | | 51 | | 68 | | 20 | | 48 | | 1000 | |
| **L1-S1-P2-1** | 0-5 | 18.1 | | 9.8 | | 123 | | 576 | | 2.7 | | 20 | | 12.7 | | 7.1 | | 0.5 | | 37 | | 192 | | 132 | | 60 | | 1000 | |
|  | 5-10 | 17.3 | | 10.3 | | 135 | | 616 | | 2.3 | | 21 | | 11.3 | | 7.4 | | 0.4 | | 39 | | 140 | | 93 | | 48 | | 1000 | |
|  | 10-20 | 18.0 | | 10.1 | | 140 | | 641 | | 1.6 | | 21 | | 10.7 | | 7.8 | | 0.4 | | 41 | | 108 | | 63 | | 45 | | 1000 | |
|  | 20-30 | 19.0 | | 10.3 | | 146 | | 657 | | 1.3 | | 22 | | 10.4 | | 8.3 | | 0.4 | | 43 | | 83 | | 39 | | 44 | | 1000 | |
|  | 30-40 | 18.2 | | 10.9 | | 156 | | 665 | | 0.9 | | 23 | | 10.2 | | 8.8 | | 0.4 | | 45 | | 63 | | 21 | | 42 | | 1000 | |
| **L1-S1-P2-2** | 0-5 | 17.9 | | 9.2 | | 118 | | 533 | | 3.1 | | 20 | | 14.0 | | 6.9 | | 0.5 | | 37 | | 240 | | 168 | | 73 | | 1000 | |
|  | 5-10 | 17.1 | | 10.8 | | 136 | | 588 | | 2.5 | | 21 | | 13.1 | | 7.4 | | 0.5 | | 40 | | 164 | | 107 | | 57 | | 1000 | |
|  | 10-20 | 18.2 | | 11.2 | | 146 | | 629 | | 1.9 | | 22 | | 12.3 | | 8.0 | | 0.5 | | 42 | | 109 | | 62 | | 47 | | 1000 | |
|  | 20-30 | 18.6 | | 10.6 | | 136 | | 614 | | 2.0 | | 22 | | 12.8 | | 7.8 | | 0.4 | | 41 | | 134 | | 81 | | 53 | | 1000 | |
|  | 30-40 | 18.6 | | 11.6 | | 157 | | 653 | | 1.3 | | 24 | | 10.8 | | 9.0 | | 0.4 | | 46 | | 68 | | 28 | | 40 | | 1000 | |
| **Slope 2** |  |  | |  | |  | |  | |  | |  | |  | |  | |  | |  | |  | |  | |  | |  | |
| **L1-S2-P1-1** | 0-5 | 16.8 | | 8.9 | | 122 | | 641 | | 2.6 | | 17 | | 16.4 | | 7.3 | | 0.5 | | 34 | | 133 | | 90 | | 43 | | 1000 | |
|  | 5-10 | 17.8 | | 9.1 | | 126 | | 670 | | 2.1 | | 18 | | 14.1 | | 7.6 | | 0.4 | | 34 | | 101 | | 62 | | 39 | | 1000 | |
|  | 10-20 | 15.8 | | 9.1 | | 123 | | 654 | | 2.4 | | 17 | | 13.7 | | 7.4 | | 0.4 | | 35 | | 122 | | 78 | | 45 | | 1000 | |
|  | 20-30 | 17.9 | | 9.7 | | 139 | | 694 | | 1.1 | | 20 | | 11.7 | | 8.2 | | 0.4 | | 36 | | 62 | | 30 | | 33 | | 1000 | |
|  | 30-40 | 20.4 | | 10.8 | | 151 | | 692 | | 0.5 | | 21 | | 11.8 | | 9.1 | | 0.4 | | 40 | | 42 | | 12 | | 30 | | 1000 | |
| **L1-S2-P1-2** | 0-5 | 18.7 | | 8.6 | | 121 | | 655 | | 2.6 | | 17 | | 15.5 | | 7.3 | | 0.5 | | 35 | | 118 | | 76 | | 42 | | 1000 | |
|  | 5-10 | 17.6 | | 9.1 | | 127 | | 669 | | 2.3 | | 17 | | 14.5 | | 7.5 | | 0.4 | | 34 | | 101 | | 64 | | 37 | | 1000 | |
|  | 10-20 | 18.2 | | 8.7 | | 121 | | 661 | | 2.5 | | 17 | | 14.1 | | 7.3 | | 0.4 | | 34 | | 116 | | 74 | | 42 | | 1000 | |
|  | 20-30 | 18.5 | | 10.0 | | 139 | | 700 | | 1.0 | | 20 | | 11.6 | | 8.2 | | 0.4 | | 37 | | 55 | | 24 | | 32 | | 1000 | |
|  | 30-40 | 21 | | 11.3 | | 150 | | 698 | | 0.4 | | 21 | | 11.5 | | 9.0 | | 0.4 | | 39 | | 39 | | 9.0 | | 29 | | 1000 | |
| **L1-S2-P2-1** | 0-5 | 18.5 | | 8.7 | | 121 | | 662 | | 2.4 | | 17 | | 13.7 | | 7.2 | | 0.4 | | 35 | | 114 | | 70 | | 44 | | 1000 | |
|  | 5-10 | 17.9 | | 8.4 | | 119 | | 663 | | 2.2 | | 17 | | 13.5 | | 7.4 | | 0.4 | | 34 | | 118 | | 72 | | 46 | | 1000 | |
|  | 10-20 | 17.7 | | 9.3 | | 130 | | 668 | | 2.1 | | 18 | | 12.2 | | 7.8 | | 0.4 | | 37 | | 99 | | 60 | | 39 | | 1000 | |
|  | 20-30 | 17.0 | | 10.2 | | 141 | | 700 | | 0.8 | | 19 | | 10.9 | | 8.4 | | 0.4 | | 40 | | 52 | | 20 | | 32 | | 1000 | |
|  | 30-40 | 19.5 | | 11.0 | | 146 | | 712 | | 0.1 | | 20 | | 12.2 | | 7.9 | | 0.5 | | 37 | | 34 | | 7.0 | | 27 | | 1000 | |
| **L1-S2-P2-2** | 0-5 | 17.6 | | 8.8 | | 124 | | 661 | | 2.4 | | 18 | | 13.4 | | 7.5 | | 0.4 | | 35 | | 112 | | 68 | | 44 | | 1000 | |
|  | 5-10 | 17.8 | | 8.4 | | 121 | | 664 | | 2.2 | | 17 | | 13.3 | | 7.4 | | 0.4 | | 35 | | 115 | | 69 | | 46 | | 1000 | |
|  | 10-20 | 19.3 | | 9.4 | | 135 | | 691 | | 1.1 | | 19 | | 10.9 | | 8.2 | | 0.4 | | 38 | | 67 | | 30 | | 37 | | 1000 | |
|  | 20-30 | 18.1 | | 11.1 | | 150 | | 696 | | 0.3 | | 21 | | 11.5 | | 9.3 | | 0.5 | | 42 | | 41 | | 9.0 | | 32 | | 1000 | |
|  | 30-40 | 20.1 | | 12.2 | | 156 | | 689 | | 0.3 | | 22 | | 11.8 | | 9.5 | | 0.5 | | 42 | | 36 | | 0 | | 36 | | 1000 | |
| **Location 2 (Ridge)** | | |  | |  | |  | |  | |  | |  | |  | |  | |  | |  | |  | |  | |  | |  |
| **Reference Site** |  |  | |  | |  | |  | |  | |  | |  | |  | |  | |  | |  | |  | |  | |  | |
| **L2-R1-P1-1** | 0-5 | 16.3 | | 8.5 | | 71.7 | | 313 | | 6.6 | | 22 | | 16 | | 4.5 | | 0.7 | | 24 | | 517 | | 397 | | 121 | | 1000 | |
|  | 5-10 | 16.2 | | 8 | | 90.3 | | 399 | | 7.3 | | 20 | | 10.9 | | 5.3 | | 0.3 | | 24 | | 419 | | 335 | | 84 | | 1000 | |
|  | 10-20 | 18.6 | | 8.8 | | 127.3 | | 503 | | 7.4 | | 21 | | 9.7 | | 7.3 | | 0.2 | | 26 | | 270 | | 195 | | 75 | | 1000 | |
|  | 20–30 | 17.1 | | 9.6 | | 157.2 | | 574 | | 9.1 | | 23 | | 9.9 | | 9.0 | | 0.2 | | 33 | | 158 | | 100 | | 58 | | 1000 | |
| **L2-R1-P1-2** | 0-5 | 15.4 | | 7.8 | | 78.4 | | 334 | | 6.8 | | 19 | | 14.1 | | 4.8 | | 0.4 | | 24 | | 495 | | 390 | | 106 | | 1000 | |
|  | 5-10 | 16.9 | | 8.4 | | 111.8 | | 431 | | 7.2 | | 19 | | 9.2 | | 6.3 | | 0.3 | | 29 | | 360 | | 269 | | 91 | | 1000 | |
|  | 10-20 | 19.8 | | 9.4 | | 141 | | 516 | | 7.3 | | 19 | | 9.5 | | 7.6 | | 0.2 | | 28 | | 243 | | 172 | | 70 | | 1000 | |
|  | 20–30 | 17.3 | | 10.1 | | 166.1 | | 603 | | 7.1 | | 23 | | 11 | | 9.6 | | 0.2 | | 31 | | 122 | | 66 | | 55 | | 1000 | |
| **L2-R1-P2-1** | 0-5 | 17.2 | | 9.4 | | 98.5 | | 433 | | 6.5 | | 26 | | 15.6 | | 6.4 | | 0.6 | | 24 | | 364 | | 281 | | 82 | | 1000 | |
|  | 5-10 | 17.1 | | 10.2 | | 131.3 | | 555 | | 6.4 | | 29 | | 10.3 | | 8.1 | | 0.3 | | 25 | | 207 | | 156 | | 51 | | 1000 | |
|  | 10-20 | 16.7 | | 10.1 | | 145.9 | | 616 | | 6.8 | | 34 | | 8.0 | | 10.5 | | 0.2 | | 23 | | 128 | | 87 | | 41 | | 1000 | |
|  | 20–30 | 17.2 | | 10 | | 155.8 | | 628 | | 5.0 | | 31 | | 9.3 | | 11.3 | | 0.2 | | 26 | | 106 | | 63 | | 43 | | 1000 | |
| **L2-R1-P2-2** | 0-5 | 18.6 | | 8.4 | | 80.6 | | 364 | | 5.9 | | 21 | | 15.1 | | 5.3 | | 0.6 | | 21 | | 460 | | 364 | | 96 | | 1000 | |
|  | 5-10 | 17.8 | | 10.1 | | 125.1 | | 530 | | 6.5 | | 30 | | 12.2 | | 8.2 | | 0.3 | | 25 | | 236 | | 179 | | 56 | | 1000 | |
|  | 10-20 | 16.2 | | 10.7 | | 149.5 | | 617 | | 5.0 | | 32 | | 7.5 | | 9.8 | | 0.2 | | 24 | | 128 | | 87 | | 41 | | 1000 | |
|  | 20–30 | 17.2 | | 10.4 | | 158.1 | | 628 | | 3.7 | | 31 | | 7.7 | | 11.1 | | 0.2 | | 25 | | 108 | | 68 | | 40 | | 1000 | |
| **Slope 3** |  |  | |  | |  | |  | |  | |  | |  | |  | |  | |  | |  | |  | |  | |  | |
| **L2-S1-P1-1** | 0-5 | 16.6 | | 9.0 | | 101.2 | | 426 | | 5.5 | | 23 | | 16 | | 6.7 | | 0.6 | | 32 | | 364 | | 274 | | 90 | | 1000 | |
|  | 5-10 | 16.6 | | 10.7 | | 137.8 | | 543 | | 4.0 | | 26 | | 11.7 | | 8.5 | | 0.3 | | 38 | | 203 | | 141 | | 62 | | 1000 | |
|  | 10-20 | 15.9 | | 11.5 | | 160.5 | | 599 | | 2.3 | | 29 | | 11.1 | | 10.4 | | 0.3 | | 46 | | 113 | | 60 | | 53 | | 1000 | |
|  | 20-30 | 15.8 | | 12.5 | | 172.2 | | 612 | | 1.5 | | 30 | | 11.5 | | 10.5 | | 0.3 | | 52 | | 83 | | 35 | | 47 | | 1000 | |
|  | 30-40 | 17.0 | | 11.8 | | 171.1 | | 612 | | 1.4 | | 30 | | 11.3 | | 10.5 | | 0.3 | | 53 | | 82 | | 32 | | 50 | | 1000 | |
| **L2-S1-P1-2** | 0-5 | 17.0 | | 8.6 | | 89.6 | | 385 | | 5.4 | | 21 | | 17.2 | | 6.0 | | 0.7 | | 29 | | 421 | | 321 | | 100 | | 1000 | |
|  | 5-10 | 15.9 | | 11.0 | | 140.9 | | 551 | | 3.7 | | 27 | | 11.9 | | 8.5 | | 0.3 | | 37 | | 193 | | 133 | | 60 | | 1000 | |
|  | 10-20 | 16.5 | | 11.2 | | 155.6 | | 598 | | 2.4 | | 29 | | 11.2 | | 10.3 | | 0.3 | | 44 | | 121 | | 68 | | 53 | | 1000 | |
|  | 20-30 | 15.9 | | 12.5 | | 170.3 | | 626 | | 1.4 | | 30 | | 11.5 | | 10.1 | | 0.3 | | 48 | | 73 | | 29 | | 45 | | 1000 | |
|  | 30-40 | 16.3 | | 12.6 | | 170.8 | | 613 | | 1.4 | | 31 | | 11.2 | | 10.8 | | 0.3 | | 53 | | 80 | | 32 | | 48 | | 1000 | |
| **L2-S1-P2-1** | 0-5 | 18.7 | | 10.1 | | 121 | | 470 | | 4.8 | | 25 | | 14.2 | | 7.6 | | 0.6 | | 39 | | 289 | | 212 | | 76 | | 1000 | |
|  | 5-10 | 18.4 | | 10.3 | | 144.5 | | 550 | | 3.4 | | 28 | | 13.1 | | 10.0 | | 0.3 | | 51 | | 172 | | 104 | | 68 | | 1000 | |
|  | 10-20 | 16.8 | | 11.3 | | 156.2 | | 584 | | 2.5 | | 27 | | 11.9 | | 10.6 | | 0.3 | | 51 | | 128 | | 71 | | 58 | | 1000 | |
|  | 20–30 | 17.8 | | 11.9 | | 166.6 | | 597 | | 1.9 | | 27 | | 11.8 | | 10.7 | | 0.3 | | 53 | | 102 | | 51 | | 52 | | 1000 | |
|  | 30-40 | – | | – | | – | | – | | – | | – | | – | | – | | – | | – | | – | | – | | – | | – | |
| **L2-S1-P2-2** | 0-5 | 18.8 | | 9.5 | | 98.5 | | 398 | | 5.3 | | 20 | | 16.1 | | 6.4 | | 0.7 | | 32 | | 395 | | 307 | | 88 | | 1000 | |
|  | 5-10 | 17.1 | | 10 | | 136.2 | | 543 | | 3.6 | | 26 | | 12.7 | | 9.5 | | 0.3 | | 47 | | 195 | | 124 | | 70 | | 1000 | |
|  | 10-20 | 17.3 | | 10.3 | | 154.4 | | 582 | | 2.6 | | 27 | | 12.5 | | 10.5 | | 0.3 | | 53 | | 131 | | 71 | | 60 | | 1000 | |
|  | 20–30 | 16.8 | | 11.9 | | 168.4 | | 591 | | 2.4 | | 27 | | 12 | | 10.7 | | 0.3 | | 54 | | 106 | | 56 | | 50 | | 1000 | |
|  | 30-40 | – | | – | | – | | – | | – | | – | | – | | – | | – | | – | | – | | – | | – | | – | |
| **Slope 4** |  |  | |  | |  | |  | |  | |  | |  | |  | |  | |  | |  | |  | |  | |  | |
| **L2-S2-P1-1** | 0-5 | 15.9 | | 11.1 | | 125.8 | | 485 | | 4.6 | | 25 | | 17.3 | | 7.1 | | 0.4 | | 34 | | 274 | | 190 | | 85 | | 1000 | |
|  | 5-10 | 16.9 | | 11.4 | | 140.3 | | 550 | | 2.9 | | 28 | | 15.4 | | 8.1 | | 0.4 | | 38 | | 189 | | 126 | | 63 | | 1000 | |
|  | 10-20 | 16.1 | | 12.3 | | 166.4 | | 616 | | 1.7 | | 32 | | 10.5 | | 9.3 | | 0.3 | | 46 | | 90 | | 44 | | 46 | | 1000 | |
|  | 20-30 | – | | – | | – | | – | | – | | – | | – | | – | | – | | – | | – | | – | | – | | – | |
| **L2-S2-P1-2** | 0-5 | 17.2 | | 10.4 | | 125.5 | | 502 | | 4.1 | | 25 | | 17 | | 7 | | 0.4 | | 32 | | 260 | | 190 | | 70 | | 1000 | |
|  | 5-10 | 14.9 | | 10.9 | | 138.9 | | 546 | | 3.2 | | 28 | | 16.6 | | 8 | | 0.4 | | 38 | | 195 | | 131 | | 65 | | 1000 | |
|  | 10-20 | 15.8 | | 12.6 | | 168.8 | | 611 | | 1.7 | | 33 | | 10.8 | | 9.4 | | 0.3 | | 45 | | 92 | | 45 | | 47 | | 1000 | |
|  | 20-30 | – | | – | | – | | – | | – | | – | | – | | – | | – | | – | | – | | – | | – | | – | |
| **L2-S2-P2-1** | 0-5 | 16.9 | | 11 | | 129.8 | | 499 | | 4.4 | | 25 | | 18.6 | | 7.3 | | 0.5 | | 34 | | 253 | | 179 | | 74 | | 1000 | |
|  | 5-10 | 15.1 | | 11.3 | | 141.9 | | 556 | | 3.1 | | 29 | | 16.6 | | 8.2 | | 0.4 | | 38 | | 180 | | 121 | | 59 | | 1000 | |
|  | 10-20 | 18 | | 12.8 | | 164.9 | | 608 | | 1.8 | | 33 | | 11.8 | | 9.4 | | 0.3 | | 45 | | 95 | | 48 | | 47 | | 1000 | |
|  | 20-30 | – | | – | | – | | – | | – | | – | | – | | – | | – | | – | | – | | – | | – | | – | |
| **L2-S2-P2-2** | 0-5 | 18 | | 11.5 | | 136.3 | | 519 | | 4.1 | | 26 | | 17.4 | | 7.6 | | 0.4 | | 34 | | 225 | | 160 | | 65 | | 1000 | |
|  | 5-10 | 16.2 | | 11.5 | | 149.4 | | 578 | | 2.9 | | 30 | | 14.6 | | 8.7 | | 0.4 | | 38 | | 151 | | 96 | | 55 | | 1000 | |
|  | 10-20 | 15.7 | | 12.4 | | 168.1 | | 617 | | 1.5 | | 33 | | 11.5 | | 9.6 | | 0.3 | | 43 | | 88 | | 42 | | 46 | | 1000 | |
|  | 20-30 | – | | – | | – | | – | | – | | – | | – | | – | | – | | – | | – | | – | | – | | – | |

Gerald Raab^a,b*^, Markus Egli^a^, Kevin P. Norton^c^, Adam P. Martin^d^, Michael E. Ketterer^e^, Dmitry Tikhomirov^a^, Rahel Wanner^f^, Fabio Scarciglia^g^

^a^ Department of Geography, University of Zurich, Winterthurerstrasse 190, 8057 Zurich, Switzerland

^b^ Department of Earth and Environmental Sciences, Dalhousie University, PO BOX 15000, 1459 Oxford Street, Halifax

^c^ School of Geography, Environment and Earth Sciences, Te Herenga Waka, Victoria University of Wellington, PO Box 600, 6140 Wellington, New Zealand

^d^ GNS Science, Private Bag 1930, Dunedin, New Zealand

^e^ Chemistry and Biochemistry, Northern Arizona University, Box 5698, Flagstaff, AZ 86011-5698, USA

^f^ Institute of Natural Resource Sciences, Zurich University of Applied Sciences, Grüental, 8820 Wädenswil, Switzerland

^g^ Department of Biology, Ecology and Earth Sciences (DiBEST), University of Calabria, Via P. Bucci – Cubo 15B, 87036 Arcavacata di Rende (CS), Italy

*Corresponding author. Tel.: +41 44 635 65 27; Fax: +41 44 6356848.

E-mail address: gr.science@gmx.at (G. Raab).
