# Supplementary material for: Soil weathering dynamics and erosion in a dry oceanic area of the southern hemisphere (Otago, New Zealand)
Source: Sci Rep. 2022 Nov 17;12:19803. doi: 10.1038/s41598-022-23731-7 (PMC9672066; doi:10.1038/s41598-022-23731-7)
Supplement: Supplementary file 7 — Supplementary Table S2. [file 41598_2022_23731_MOESM7_ESM.doc]

**Table S2**: Trace element content of soils in ppm (parts per million)

|  | Depth | S | V | Cr | Co | Ni | Cu | Zn | Ga | Ge | As | Se | Br | Rb | Sr | Y | Zr | Nb | Mo | Cd | Sn | I | Cs | Ba | La | Ce | Pr | Nd | Hf | W | Pb | Th | U |
| --- | --- | --- | --- | --- | --- | --- | --- | --- | --- | --- | --- | --- | --- | --- | --- | --- | --- | --- | --- | --- | --- | --- | --- | --- | --- | --- | --- | --- | --- | --- | --- | --- | --- |
|  | (cm) | ppm | ppm | ppm | ppm | ppm | ppm | ppm | ppm | ppm | ppm | ppm | ppm | ppm | ppm | ppm | ppm | ppm | ppm | ppm | ppm | ppm | ppm | ppm | ppm | ppm | ppm | ppm | ppm | ppm | ppm | ppm | ppm |
| **Location 1 (Valley** | | | | | |  |  |  |  |  |  |  |  |  |  |  |  |  |  |  |  |  |  |  |  |  |  |  |  |  |  |  |  |
| **Reference Site** | | | | | |  |  |  |  |  |  |  |  |  |  |  |  |  |  |  |  |  |  |  |  |  |  |  |  |  |  |  |  |
| **L1-R1-P1-1** | 0-5 | 1198 | 59 | 32 | 13.3 | 14.9 | 7.8 | 48 | 14.7 | 0.5 | 6.1 | – | 11.2 | 84 | 187 | 11.5 | 183 | 6.0 | 2.6 | 0.2 | – | – | 7.5 | 286 | 26 | 48 | 39 | 49 | 5.9 | 43 | 12.9 | 7.6 | 2.4 |
|  | 5-10 | 800 | 69 | 31 | 7.9 | 14.2 | 5.0 | 44 | 16.7 | 0.9 | 6.8 | – | 11.6 | 91 | 200 | 12.8 | 205 | 6.8 | 2.5 | – | – | – | 8.4 | 297 | 28 | 51 | 43 | 58 | 4.6 | 16 | 14.4 | 8.3 | 3.0 |
|  | 10-20 | 515 | 63 | 36 | 7.1 | 14.6 | 7.7 | 43 | 15.7 | 0.5 | 7.5 | – | 13.9 | 93 | 204 | 13.8 | 217 | 7.1 | 2.1 | – | – | – | 8.4 | 310 | 26 | 48 | 31 | 46 | 7.3 | 35 | 15.1 | 8.8 | 3.5 |
|  | 20–25 | 428 | 72 | 33 | 13.3 | 14.3 | 6.0 | 43 | 16.7 | 0.9 | 7.1 | – | 14.6 | 92 | 206 | 14.0 | 214 | 6.7 | 2.2 | – | – | – | 9.4 | 321 | 31 | 54 | 45 | 61 | 4.8 | 39 | 15.7 | 9.3 | 3.3 |
| **L1-R1-P1-2** | 0-5 | 1328 | 58 | 32 | 19.7 | 16.8 | 7.8 | 48 | 14.9 | 0.0 | 5.6 | – | 11.3 | 84 | 185 | 11.1 | 188 | 5.7 | 2.1 | – | – | – | 8.8 | 288 | 30 | 46 | 44 | 57 | 5.6 | 58 | 13.4 | 7.2 | 2.2 |
|  | 5-10 | 928 | 71 | 33 | 6.5 | 14.1 | 6.5 | 43 | 16.7 | 0.5 | 6.3 | – | 11.6 | 88 | 193 | 12.3 | 206 | 6.6 | 1.5 | – | – | – | 12.2 | 290 | 35 | 56 | 53 | 59 | 4.8 | 18 | 14.0 | 7.8 | 2.8 |
|  | 10-20 | 643 | 66 | 36 | 8.1 | 13.8 | 4.6 | 43 | 16.1 | 0.8 | 6.6 | – | 13.1 | 90 | 199 | 13.4 | 215 | 7.0 | 1.7 | – | – | 8.9 | 7.3 | 308 | 25 | 45 | 31 | 43 | 6.1 | 12 | 14.7 | 8.5 | 3.0 |
|  | 20–25 | 444 | 70 | 33 | 11.3 | 13.6 | 5.0 | 44 | 16.4 | 0.8 | 7.6 | – | 14.1 | 91 | 202 | 13.9 | 213 | 6.7 | 1.5 | – | – | – | 12.9 | 316 | 34 | 58 | 53 | 66 | 6.0 | 29 | 15.0 | 9.1 | 3.2 |
| **L1-R1-P2-1** | 0-5 | 1640 | 59 | 26 | 10.3 | 13.2 | 5.9 | 42 | 13.9 | 0.6 | 6.2 | – | 12.2 | 83 | 168 | 10.3 | 157 | 5.7 | 1.9 | – | – | – | 14.2 | 294 | 38 | 57 | 66 | 82 | 5.5 | 29 | 11.6 | 6.4 | 2.5 |
|  | 5-10 | 725 | 63 | 30 | 6.2 | 14.4 | 3.7 | 36 | 15.9 | 0.0 | 6.9 | – | 10.9 | 88 | 185 | 11.9 | 175 | 6.4 | 1.6 | – | – | – | 9.6 | 303 | 30 | 54 | 49 | 58 | 4.4 | 44 | 12.7 | 7.7 | 3.7 |
|  | 10-20 | 508 | 70 | 31 | – | 13.4 | 4.2 | 38 | 17.7 | 1.1 | 7.6 | – | 13.6 | 93 | 192 | 13.1 | 190 | 7.0 | 1.7 | – | – | – | 10.2 | 314 | 30 | 55 | 50 | 63 | 4.1 | 11 | 13.3 | 8.0 | 3.1 |
|  | 20–25 | 395 | 64 | 35 | – | 14.1 | 5.7 | 38 | 17.8 | 0.9 | 8.1 | – | 13.0 | 93 | 197 | 13.3 | 185 | 6.8 | 2.0 | – | – | – | 8.5 | 327 | 27 | 49 | 41 | 55 | 4.7 | 9 | 13.9 | 8.3 | 2.7 |
| **L1-R1-P2-2** | 0-5 | 1668 | 60 | 26 | 8.9 | 13.1 | 5.7 | 42 | 13.4 | 0.6 | 5.9 | – | 10.8 | 81 | 167 | 9.7 | 153 | 5.5 | 2.7 | 0.3 | 0.8 | – | 17.8 | 287 | 41 | 63 | 70 | 83 | 5.1 | 32 | 11.3 | 6.5 | 2.0 |
|  | 5-10 | 773 | 67 | 29 | 5.2 | 13.9 | 5.1 | 37 | 16.4 | 0.8 | 7.0 | – | 11.3 | 91 | 186 | 11.8 | 194 | 6.6 | 1.8 | – | – | – | 9.9 | 306 | 30 | 51 | 46 | 58 | 3.8 | 25 | 13.1 | 7.4 | 2.5 |
|  | 10-20 | 528 | 63 | 33 | 9.4 | 12.3 | 4.2 | 37 | 16.8 | 0.9 | 6.7 | – | 13.3 | 90 | 194 | 12.9 | 202 | 7.0 | 1.3 | – | – | – | 7.3 | 328 | 27 | 50 | 36 | 56 | 5.2 | 7 | 14.2 | 7.9 | 3.0 |
|  | 20–25 | 505 | 71 | 31 | 6.8 | 12.9 | 5.8 | 41 | 18.1 | 0.7 | 7.3 | – | 12.6 | 96 | 206 | 13.4 | 193 | 6.6 | 2.1 | – | – | – | 10.2 | 367 | 30 | 53 | 46 | 58 | 3.6 | 6 | 15.5 | 8.6 | 3.1 |
| **Slope 1** | | | | | |  |  |  |  |  |  |  |  |  |  |  |  |  |  |  |  |  |  |  |  |  |  |  |  |  |  |  |  |
| **L1-S1-P1-1** | 0-5 | 1239 | 73 | 31 | 9.9 | 14.7 | 6.1 | 43 | 17.1 | 0.7 | 6.7 | – | 18.5 | 81 | 207 | 13.1 | 195 | 6.6 | 1.8 | – | 0.5 | – | 15.4 | 307 | 43 | 69 | 65 | 84 | 3.6 | 37 | 14.5 | 8.6 | 2.6 |
|  | 5-10 | 796 | 83 | 36 | 6.8 | 16.8 | 6.6 | 44 | 17.6 | 1.0 | 7.7 | – | 22.1 | 88 | 223 | 15.0 | 209 | 6.9 | 2.4 | – | 0.5 | – | 12.4 | 310 | 37 | 63 | 56 | 65 | 6.5 | 8 | 15.3 | 10.1 | 3.3 |
|  | 10-20 | 655 | 84 | 36 | 11.5 | 15.8 | 4.3 | 45 | 17.7 | 0.7 | 8.2 | – | 23.7 | 89 | 229 | 16.3 | 227 | 7.5 | 1.7 | 0.2 | 0.0 | – | 9.7 | 327 | 31 | 62 | 50 | 63 | 6.7 | 15 | 16.3 | 10.5 | 4.1 |
|  | 20-30 | 506 | 78 | 37 | 12.7 | 16.8 | 4.5 | 46 | 19.1 | 1.1 | 8.3 | 0.1 | 26.7 | 88 | 236 | 17.5 | 232 | 7.6 | 1.9 | 0.4 | 1.4 | 27.1 | 19.2 | 331 | 50 | 84 | 84 | 97 | 5.8 | 8 | 17.4 | 11.8 | 3.7 |
|  | 30-40 | 304 | 90 | 44 | 11.0 | 17.3 | 5.7 | 51 | 18.9 | 0.6 | 10.3 | 0.2 | 27.5 | 88 | 251 | 19.7 | 264 | 9.2 | 1.4 | – | – | 35.1 | 9.0 | 327 | 33 | 68 | 42 | 59 | 8.2 | 7 | 19.7 | 12.7 | 4.0 |
| **L1-S1-P1-2** | 0-5 | 1315 | 71 | 31 | 13.9 | 15.0 | 6.3 | 44 | 16.4 | 0.0 | 6.8 | – | 18.1 | 82 | 208 | 12.9 | 196 | 6.3 | 2.1 | – | – | – | 9.9 | 301 | 34 | 58 | 50 | 65 | 5.3 | 12 | 14.9 | 8.9 | 2.9 |
|  | 5-10 | 822 | 81 | 35 | 8.0 | 14.3 | 5.5 | 45 | 17.5 | 0.9 | 7.7 | 0.2 | 21.6 | 91 | 221 | 14.7 | 215 | 7.2 | 1.4 | – | – | – | 8.1 | 329 | 29 | 56 | 40 | 58 | 5.5 | 4 | 16.2 | 10.3 | 3.0 |
|  | 10-20 | 509 | 85 | 37 | 9.8 | 15.2 | 4.6 | 45 | 18.2 | 0.8 | 8.3 | 0.2 | 28.6 | 90 | 231 | 16.1 | 224 | 7.8 | 1.6 | 0.4 | – | 24.5 | 8.4 | 331 | 32 | 59 | 41 | 60 | 6.1 | 8 | 16.5 | 11.0 | 3.6 |
|  | 20-30 | 411 | 85 | 40 | 9.7 | 17.7 | 7.3 | 46 | 19.2 | 0.7 | 8.6 | 0.3 | 36.5 | 86 | 235 | 18.0 | 236 | 8.2 | 2.2 | 0.2 | – | 34.6 | 10.5 | 315 | 32 | 65 | 43 | 64 | 6.6 | 11 | 17.5 | 11.9 | 3.6 |
|  | 30-40 | 293 | 90 | 44 | 14.6 | 20.4 | 8.2 | 53 | 19.8 | 0.9 | 10.4 | 0.2 | 30.5 | 86 | 245 | 20.0 | 256 | 9.4 | 1.5 | – | – | 36.5 | 7.0 | 335 | 30 | 64 | 37 | 52 | 5.5 | 9 | 20.3 | 13.6 | 3.7 |
| **L1-S1-P2-1** | 0-5 | 1089 | 73 | 33 | 10.0 | 16.0 | 5.8 | 47 | 14.9 | 0.8 | 7.3 | – | 18.3 | 77 | 205 | 13.1 | 195 | 6.5 | 1.7 | – | – | – | 7.3 | 282 | 30 | 52 | 39 | 47 | 6.9 | 11 | 13.7 | 8.9 | 2.3 |
|  | 5-10 | 944 | 78 | 35 | 9.9 | 17.9 | 8.3 | 47 | 17.3 | 0.7 | 7.6 | – | 20.6 | 80 | 213 | 14.6 | 212 | 6.8 | 2.6 | 0.2 | – | – | 10.0 | 291 | 32 | 57 | 47 | 57 | 6.2 | 9 | 14.6 | 9.4 | 2.8 |
|  | 10-20 | 590 | 79 | 37 | 6.8 | 17.0 | 4.9 | 46 | 16.7 | 1.2 | 7.8 | 0.2 | 24.2 | 80 | 216 | 15.3 | 224 | 7.3 | 1.8 | – | – | – | 8.3 | 302 | 32 | 58 | 45 | 61 | 6.5 | 3 | 14.9 | 10.2 | 3.2 |
|  | 20-30 | 420 | 83 | 40 | 12.1 | 16.7 | 5.0 | 48 | 18.9 | 1.1 | 8.4 | 0.2 | 30.1 | 83 | 226 | 16.8 | 232 | 7.7 | 1.4 | 0.2 | 1.3 | 26.2 | 13.5 | 312 | 41 | 68 | 61 | 73 | 5.5 | 9 | 16.1 | 10.6 | 3.3 |
|  | 30-40 | 303 | 83 | 40 | 7.4 | 19.4 | 5.5 | 51 | 17.7 | 0.8 | 9.2 | 0.1 | 27.5 | 84 | 235 | 18.7 | 255 | 8.5 | 1.7 | 0.3 | – | 29.0 | 11.4 | 320 | 40 | 70 | 54 | 76 | 7.8 | 8 | 17.7 | 11.8 | 4.0 |
| **L1-S1-P2-2** | 0-5 | 1361 | 69 | 37 | 11.3 | 16.3 | 6.7 | 47 | 15.0 | 0.6 | 7.1 | – | 19.7 | 73 | 196 | 12.5 | 179 | 6.0 | 2.3 | 0.3 | – | – | 9.2 | 278 | 29 | 49 | 43 | 55 | 5.8 | 20 | 13.5 | 8.2 | 2.5 |
|  | 5-10 | 1009 | 74 | 33 | 10.3 | 16.2 | 4.3 | 45 | 15.8 | 0.5 | 7.8 | – | 21.4 | 80 | 216 | 14.2 | 194 | 7.1 | 1.9 | – | – | – | 10.3 | 324 | 35 | 58 | 47 | 63 | 6.8 | 13 | 14.8 | 9.7 | 3.3 |
|  | 10-20 | 648 | 79 | 36 | 4.9 | 16.2 | 4.8 | 46 | 17.1 | 0.7 | 8.6 | 0.1 | 23.3 | 82 | 227 | 15.6 | 224 | 7.2 | 2.0 | 0.3 | – | – | 11.8 | 312 | 35 | 60 | 51 | 66 | 6.5 | 9 | 15.6 | 10.3 | 3.7 |
|  | 20-30 | 735 | 78 | 34 | 11.2 | 16.6 | 6.3 | 45 | 17.5 | 0.0 | 7.1 | 0.2 | 21.5 | 80 | 218 | 14.5 | 205 | 6.8 | 1.8 | 0.3 | – | – | 9.4 | 330 | 32 | 58 | 46 | 63 | 5.2 | 5 | 15.2 | 9.7 | 3.2 |
|  | 30-40 | 351 | 83 | 41 | 14.5 | 17.1 | 6.7 | 50 | 18.6 | 0.5 | 9.4 | 0.2 | 28.0 | 84 | 231 | 17.4 | 229 | 8.2 | 1.6 | – | – | 21.1 | 7.1 | 325 | 28 | 57 | 35 | 56 | 5.8 | 7 | 17.0 | 11.5 | 4.1 |
| **Slope 2** | | | | | |  |  |  |  |  |  |  |  |  |  |  |  |  |  |  |  |  |  |  |  |  |  |  |  |  |  |  |  |
| **L1-S2-P1-1** | 0-5 | 838 | 70 | 33 | 5.8 | 15.4 | 3.6 | 39 | 14.6 | 0.7 | 7.1 | – | 17.8 | 65 | 206 | 14.7 | 226 | 6.6 | 1.6 | 0.3 | – | – | 10.6 | 250 | 33 | 59 | 51 | 59 | 6.7 | 15 | 13.0 | 8.1 | 2.6 |
|  | 5-10 | 733 | 72 | 33 | 5.5 | 15.0 | 1.8 | 39 | 14.9 | 0.8 | 6.8 | 0.1 | 19.2 | 66 | 215 | 15.5 | 247 | 7.1 | 1.4 | – | 0.5 | – | 10.1 | 255 | 33 | 58 | 43 | 59 | 9.3 | 4 | 13.1 | 8.4 | 3.3 |
|  | 10-20 | 854 | 73 | 33 | 10.0 | 16.2 | 4.5 | 40 | 15.3 | 0.0 | 6.8 | – | 18.5 | 65 | 209 | 15.0 | 226 | 6.9 | 1.9 | 0.3 | – | – | 10.7 | 255 | 36 | 57 | 54 | 64 | 5.5 | 11 | 13.7 | 8.2 | 3.4 |
|  | 20-30 | 418 | 76 | 36 | 6.2 | 15.8 | 3.4 | 41 | 17.0 | 0.8 | 7.1 | 0.2 | 17.9 | 70 | 232 | 17.5 | 261 | 7.4 | 1.3 | – | – | – | 7.2 | 272 | 29 | 53 | 38 | 55 | 5.5 | 4 | 13.5 | 9.1 | 3.5 |
|  | 30-40 | 286 | 79 | 43 | 6.2 | 16.9 | 3.3 | 45 | 16.8 | 0.8 | 7.2 | – | 10.2 | 71 | 243 | 18.6 | 275 | 8.3 | 2.0 | 0.2 | – | – | 10.4 | 295 | 37 | 70 | 53 | 66 | 7.7 | 8 | 15.0 | 10.2 | 3.5 |
| **L1-S2-P1-2** | 0-5 | 784 | 70 | 33 | 9.7 | 16.9 | 6.1 | 40 | 16.3 | 0.0 | 7.0 | – | 17.6 | 65 | 209 | 15.3 | 256 | 6.9 | 1.8 | 0.2 | – | – | 9.3 | 258 | 32 | 56 | 46 | 59 | 6.0 | 16 | 13.3 | 8.3 | 3.1 |
|  | 5-10 | 763 | 73 | 34 | 4.5 | 14.5 | 4.7 | 40 | 15.7 | 0.5 | 7.5 | 0.2 | 18.9 | 65 | 209 | 14.9 | 232 | 7.0 | 1.9 | – | – | 13.8 | 9.3 | 252 | 32 | 58 | 49 | 66 | 6.4 | 6 | 13.1 | 8.2 | 4.1 |
|  | 10-20 | 841 | 72 | 32 | 7.9 | 15.3 | 3.8 | 39 | 15.5 | 0.8 | 6.8 | 0.1 | 18.2 | 63 | 205 | 14.6 | 236 | 7.1 | 2.1 | – | – | – | 9.1 | 255 | 31 | 54 | 43 | 66 | 5.2 | 4 | 13.1 | 8.1 | 3.1 |
|  | 20-30 | 404 | 76 | 37 | – | 16.1 | 3.7 | 40 | 17.3 | 1.0 | 6.4 | – | 15.9 | 69 | 235 | 17.8 | 275 | 7.3 | 1.3 | – | – | – | 8.5 | 276 | 33 | 63 | 49 | 67 | 6.1 | 6 | 14.2 | 9.0 | 3.9 |
|  | 30-40 | 263 | 78 | 39 | 5.1 | 17.1 | 5.2 | 44 | 17.7 | 0.8 | 7.6 | – | 8.9 | 69 | 248 | 19.3 | 290 | 7.8 | – | – | – | – | 5.8 | 300 | 30 | 63 | 36 | 58 | 7.6 | 9 | 14.7 | 10.3 | 3.9 |
| **L1-S2-P2-1** | 0-5 | 784 | 71 | 32 | 8.9 | 14.6 | 4.7 | 39 | 15.8 | 0.0 | 7.4 | – | 19.0 | 64 | 210 | 14.8 | 246 | 7.1 | 1.1 | – | – | 14.3 | 7.7 | 259 | 32 | 55 | 40 | 58 | 4.7 | 6 | 12.9 | 8.4 | 3.2 |
|  | 5-10 | 762 | 70 | 32 | – | 14.3 | 4.8 | 39 | 15.6 | 0.8 | 7.1 | 0.2 | 20.7 | 64 | 209 | 15.1 | 238 | 7.4 | 1.1 | 0.3 | – | – | 4.6 | 258 | 25 | 50 | 34 | 51 | 6.5 | 2 | 13.5 | 8.3 | 3.6 |
|  | 10-20 | 745 | 66 | 32 | – | 14.9 | 2.3 | 41 | 15.7 | 0.7 | 7.7 | 0.2 | 21.1 | 67 | 214 | 15.9 | 235 | 7.5 | 1.8 | 0.7 | 3.7 | 30.9 | 24.4 | 275 | 59 | 92 | 105 | 126 | 5.7 | 6 | 14.0 | 8.8 | 3.3 |
|  | 20-30 | 339 | 76 | 38 | 6.5 | 16.5 | 2.9 | 42 | 17.1 | 0.0 | 7.8 | – | 17.4 | 69 | 230 | 17.8 | 258 | 7.3 | 1.2 | – | – | – | 5.4 | 270 | 27 | 53 | 30 | 47 | 6.5 | 6 | 14.9 | 9.7 | 3.5 |
|  | 30-40 | 189 | 74 | 38 | 5.2 | 17.4 | 5.9 | 43 | 17.0 | 0.4 | 6.4 | – | 7.3 | 60 | 254 | 19.4 | 302 | 7.0 | 0.9 | – | – | – | 7.4 | 278 | 32 | 67 | 45 | 65 | 5.8 | 8 | 14.6 | 10.2 | 4.6 |
| **L1-S2-P2-2** | 0-5 | 789 | 69 | 32 | 8.5 | 15.3 | 3.7 | 39 | 15.8 | 0.6 | 7.3 | 0.1 | 19.9 | 65 | 207 | 15.3 | 228 | 6.7 | 1.2 | 0.2 | – | – | 7.6 | 252 | 32 | 55 | 43 | 58 | 5.6 | 6 | 13.0 | 8.2 | 4.1 |
|  | 5-10 | 761 | 66 | 38 | 10.5 | 15.3 | 5.4 | 40 | 15.9 | 0.5 | 7.4 | 0.3 | 20.9 | 64 | 210 | 15.0 | 231 | 7.3 | 1.9 | 0.2 | – | – | 5.6 | 252 | 26 | 49 | 31 | 47 | 6.2 | 2 | 13.2 | 8.8 | 4.2 |
|  | 10-20 | 386 | 76 | 36 | 7.2 | 15.1 | 3.2 | 40 | 16.9 | 0.9 | 8.0 | 0.1 | 22.5 | 69 | 221 | 16.9 | 245 | 7.5 | 1.9 | 0.3 | 0.9 | – | 11.4 | 264 | 37 | 63 | 53 | 67 | 6.6 | 10 | 14.4 | 9.2 | 3.3 |
|  | 20-30 | 227 | 80 | 40 | – | 16.8 | 2.3 | 47 | 17.0 | 0.8 | 8.0 | – | 9.3 | 70 | 244 | 19.4 | 287 | 8.0 | 1.5 | – | – | – | 8.4 | 293 | 31 | 63 | 42 | 65 | 8.4 | 8 | 15.6 | 10.9 | 3.9 |
|  | 30-40 | 163 | 81 | 47 | 12.6 | 18.5 | 7.8 | 49 | 18.1 | 0.9 | 7.1 | – | 5.0 | 71 | 251 | 20.7 | 299 | 8.4 | 1.2 | – | – | – | 6.3 | 326 | 33 | 69 | 44 | 64 | 7.4 | 5 | 15.9 | 11.1 | 3.7 |
| **Location 2 (Ridge)** | | | |  |  |  |  |  |  |  |  |  |  |  |  |  |  |  |  |  |  |  |  |  |  |  |  |  |  |  |  |  |  |
| **Reference Site** | | | | | |  |  |  |  |  |  |  |  |  |  |  |  |  |  |  |  |  |  |  |  |  |  |  |  |  |  |  |  |
| **L2-R1-P1-1** | 0-5 | 3880 | 56 | 25 | 14.4 | 13.6 | 14.7 | 84 | 11.2 | – | 2.1 | 0.1 | 26.1 | 68 | 140 | 9.4 | 101 | 4.7 | 3.5 | – | – | – | 11.5 | 238 | 34 | 53 | 54 | 65 | 4.2 | 21 | 12.3 | 6.2 | 1.7 |
|  | 5-10 | 3212 | 67 | 30 | 12.9 | 16.3 | 14.5 | 57 | 13.5 | – | 2.7 | – | 28.7 | 77 | 152 | 12.2 | 122 | 5.6 | 2.5 | 0.2 | – | 19.4 | 16.0 | 264 | 43 | 65 | 62 | 77 | 4.3 | 62 | 14.3 | 8.2 | 2.5 |
|  | 10-20 | 1832 | 90 | 37 | 12.6 | 15.3 | 11.1 | 33 | 17.6 | 1.0 | 3.0 | 0.7 | 50.6 | 67 | 183 | 19.3 | 154 | 7.0 | 1.4 | – | – | 29.9 | 6.5 | 255 | 39 | 72 | 38 | 62 | 4.7 | 5 | 18.4 | 10.3 | 3.4 |
|  | 20–30 | 1061 | 106 | 42 | 6.0 | 13.0 | 12.0 | 28 | 19.7 | 1.1 | 4.2 | 0.8 | 64.0 | 69 | 203 | 21.9 | 206 | 8.5 | 1.9 | – | 0.8 | 37.9 | 10.4 | 271 | 48 | 85 | 50 | 73 | 7.2 | 4 | 22.6 | 11.5 | 3.7 |
| **L2-R1-P1-2** | 0-5 | 3558 | 62 | 27 | 18.6 | 17.6 | 15.6 | 59 | 12.1 | 0.4 | 2.7 | – | 30.7 | 68 | 142 | 10.7 | 105 | 4.7 | 3.2 | – | – | – | 10.9 | 244 | 35 | 55 | 44 | 56 | 4.2 | 57 | 13.5 | 7.2 | 2.0 |
|  | 5-10 | 2591 | 77 | 35 | 17.5 | 18.5 | 15.0 | 39 | 15.3 | 0.5 | 3.6 | 0.1 | 50.3 | 75 | 168 | 16.4 | 138 | 6.9 | 2.2 | 0.2 | – | 30.7 | 10.0 | 255 | 40 | 68 | 50 | 63 | 4.9 | 35 | 18.6 | 10.1 | 3.3 |
|  | 10-20 | 1600 | 86 | 38 | 9.7 | 14.9 | 14.3 | 34 | 17.7 | 0.8 | 4.1 | 0.8 | 66.0 | 65 | 186 | 21.0 | 161 | 7.3 | 1.7 | – | 0.7 | 41.4 | 13.5 | 246 | 52 | 87 | 59 | 77 | 5.8 | 6 | 21.6 | 11.6 | 3.5 |
|  | 20–30 | 638 | 109 | 45 | – | 12.4 | 13.6 | 30 | 22.1 | 1.0 | 3.7 | 0.8 | 58.6 | 70 | 228 | 22.4 | 239 | 9.4 | 2.0 | – | 1.2 | 41.8 | 11.0 | 288 | 48 | 90 | 46 | 72 | 8.1 | 4 | 23.5 | 12.1 | 3.8 |
| **L2-R1-P2-1** | 0-5 | 2939 | 78 | 34 | 10.0 | 12.9 | 11.2 | 69 | 14.7 | – | 2.1 | – | 16.2 | 92 | 157 | 9.6 | 152 | 6.1 | 3.4 | – | 0.0 | 14.4 | 13.1 | 320 | 35 | 54 | 57 | 64 | 4.6 | 11 | 12.6 | 7.0 | 2.2 |
|  | 5-10 | 1588 | 94 | 40 | 10.3 | 12.7 | 8.3 | 41 | 19.5 | – | 2.5 | – | 17.9 | 102 | 166 | 11.7 | 193 | 8.0 | 2.0 | 0.3 | – | – | 12.1 | 332 | 32 | 52 | 49 | 55 | 4.2 | 43 | 14.5 | 8.3 | 2.9 |
|  | 10-20 | 1035 | 108 | 50 | – | 11.4 | 3.8 | 31 | 21.6 | 1.4 | 2.9 | – | 14.5 | 99 | 152 | 10.7 | 229 | 8.8 | 2.9 | 0.2 | – | – | 10.9 | 323 | 26 | 42 | 42 | 48 | 6.0 | 13 | 14.2 | 7.1 | 2.9 |
|  | 20–30 | 773 | 112 | 46 | – | 11.2 | 6.6 | 27 | 23.3 | 1.6 | 3.0 | 0.3 | 32.1 | 86 | 183 | 14.3 | 269 | 10.0 | 1.9 | – | 1.3 | 22.8 | 12.8 | 328 | 35 | 57 | 47 | 63 | 5.6 | 7 | 18.8 | 8.8 | 3.1 |
| **L2-R1-P2-2** | 0-5 | 3545 | 61 | 32 | 12.1 | 12.7 | 14.3 | 83 | 12.8 | – | 1.8 | 0.1 | 20.2 | 78 | 138 | 8.3 | 124 | 5.5 | 2.9 | 0.3 | – | – | 10.6 | 278 | 31 | 46 | 46 | 50 | 5.2 | 20 | 11.3 | 5.7 | 2.3 |
|  | 5-10 | 1798 | 91 | 39 | 5.6 | 12.9 | 11.3 | 54 | 18.3 | 0.8 | 2.6 | – | 12.8 | 107 | 165 | 11.1 | 190 | 7.7 | 2.7 | 0.3 | – | – | 11.6 | 342 | 31 | 50 | 55 | 63 | 4.9 | 34 | 14.4 | 7.6 | 2.9 |
|  | 10-20 | 913 | 104 | 50 | – | 13.3 | 7.4 | 32 | 20.9 | 1.2 | 2.8 | 0.2 | 20.7 | 86 | 151 | 11.7 | 228 | 8.6 | 1.6 | – | 1.3 | – | 10.7 | 337 | 28 | 45 | 39 | 50 | 5.4 | 10 | 14.7 | 7.1 | 2.8 |
|  | 20–30 | 664 | 113 | 47 | – | 10.9 | 3.7 | 28 | 23.3 | 1.7 | 3.0 | 0.4 | 32.0 | 89 | 155 | 12.4 | 267 | 9.5 | 2.6 | – | 1.2 | – | 12.7 | 354 | 31 | 52 | 50 | 62 | 8.1 | 5 | 17.4 | 7.9 | 2.6 |
| **Slope 3** | | | | | |  |  |  |  |  |  |  |  |  |  |  |  |  |  |  |  |  |  |  |  |  |  |  |  |  |  |  |  |
| **L2-S1-P1-1** | 0-5 | 2788 | 82 | 31 | 10.8 | 13.9 | 9.6 | 62 | 15.7 | 0.6 | 2.7 | – | 14.6 | 99 | 191 | 11.1 | 149 | 6.2 | 3.0 | 0.4 | – | – | 10.6 | 283 | 30 | 52 | 51 | 63 | 5.1 | 15 | 15.8 | 7.9 | 3.1 |
|  | 5-10 | 1399 | 97 | 36 | 8.2 | 13.5 | 4.6 | 43 | 19.1 | 0.7 | 3.8 | – | 15.6 | 117 | 225 | 14.3 | 203 | 8.0 | 1.9 | – | – | – | 14.9 | 327 | 41 | 66 | 65 | 79 | 6.5 | 29 | 19.7 | 10.0 | 3.6 |
|  | 10-20 | 607 | 114 | 43 | 10.7 | 13.6 | 5.2 | 43 | 22.6 | 0.9 | 4.8 | 0.1 | 18.2 | 123 | 251 | 17.2 | 221 | 8.9 | 1.8 | – | – | – | 10.7 | 344 | 33 | 61 | 48 | 63 | 6.2 | 9 | 21.9 | 11.4 | 4.5 |
|  | 20-30 | 412 | 111 | 45 | 10.6 | 13.7 | 3.9 | 43 | 22.2 | 0.9 | 5.3 | 0.2 | 19.2 | 120 | 260 | 18.8 | 215 | 9.5 | 2.3 | – | – | 19.0 | 14.6 | 345 | 40 | 70 | 53 | 77 | 6.4 | 5 | 24.5 | 12.4 | 4.5 |
|  | 30-40 | 409 | 109 | 44 | 6.4 | 14.9 | 6.3 | 42 | 22.8 | 1.0 | 5.7 | 0.3 | 19.3 | 120 | 263 | 18.7 | 224 | 9.9 | 2.2 | – | – | – | 8.3 | 326 | 32 | 58 | 37 | 56 | 5.7 | 9 | 24.0 | 12.3 | 4.3 |
| **L2-S1-P1-2** | 0-5 | 3447 | 71 | 35 | 11.1 | 14.1 | 9.5 | 65 | 14.3 | 0.5 | 3.0 | – | 14.8 | 94 | 180 | 10.2 | 131 | 5.5 | 2.1 | 0.3 | – | – | 10.5 | 276 | 29 | 48 | 44 | 55 | 6.1 | 28 | 14.4 | 7.1 | 3.0 |
|  | 5-10 | 1276 | 98 | 38 | 8.5 | 14.3 | 5.0 | 44 | 20.5 | 1.2 | 3.5 | – | 13.9 | 120 | 231 | 14.6 | 205 | 8.2 | 1.8 | 0.3 | 0.9 | – | 16.4 | 339 | 41 | 67 | 64 | 82 | 5.4 | 36 | 19.3 | 10.4 | 3.4 |
|  | 10-20 | 725 | 116 | 44 | 4.3 | 13.1 | 3.9 | 40 | 21.6 | 1.0 | 4.1 | 0.1 | 17.5 | 120 | 246 | 16.7 | 213 | 9.1 | 2.2 | – | – | – | 10.1 | 328 | 32 | 61 | 39 | 61 | 5.6 | 9 | 21.2 | 11.0 | 4.6 |
|  | 20-30 | 390 | 107 | 44 | 6.0 | 14.1 | 4.0 | 41 | 21.1 | 1.1 | 4.8 | 0.1 | 14.0 | 116 | 253 | 17.9 | 219 | 8.6 | 2.1 | 0.3 | – | – | 10.6 | 338 | 36 | 64 | 49 | 68 | 5.5 | 10 | 22.4 | 11.7 | 4.5 |
|  | 30-40 | 404 | 115 | 47 | 11.6 | 14.6 | 3.6 | 43 | 22.5 | 0.6 | 5.3 | – | 17.4 | 126 | 260 | 19.0 | 226 | 10.0 | 1.6 | – | – | – | 9.5 | 355 | 31 | 59 | 37 | 57 | 6.6 | 10 | 24.3 | 12.2 | 4.7 |
| **L2-S1-P2-1** | 0-5 | 2108 | 80 | 39 | 15.6 | 15.0 | 10.1 | 57 | 17.9 | 0.9 | 3.7 | – | 13.2 | 104 | 212 | 12.3 | 170 | 6.9 | 2.3 | – | – | – | 8.9 | 303 | 30 | 49 | 46 | 51 | 3.6 | 29 | 17.8 | 8.7 | 3.5 |
|  | 5-10 | 1107 | 104 | 40 | 9.6 | 14.1 | 5.1 | 49 | 20.2 | 1.2 | 5.3 | 0.1 | 15.9 | 116 | 245 | 15.3 | 220 | 9.4 | 1.9 | – | 1.6 | 18.2 | 11.3 | 333 | 35 | 65 | 57 | 79 | 6.1 | 9 | 21.2 | 10.8 | 3.7 |
|  | 10-20 | 683 | 104 | 42 | 4.4 | 14.9 | 5.2 | 45 | 20.6 | 1.1 | 5.5 | 0.2 | 18.3 | 114 | 257 | 16.9 | 230 | 9.5 | 1.4 | – | – | 12.9 | 8.1 | 335 | 30 | 57 | 36 | 55 | 7.3 | 3 | 20.8 | 11.3 | 4.5 |
|  | 20–30 | 536 | 106 | 44 | 7.2 | 16.1 | 5.2 | 46 | 21.9 | 1.1 | 5.9 | 0.2 | 19.7 | 117 | 266 | 18.4 | 235 | 9.5 | 2.3 | – | – | – | 6.8 | 326 | 27 | 57 | 29 | 49 | 7.0 | 6 | 23.8 | 12.1 | 4.3 |
| **L2-S1-P2-2** | 0-5 | 3293 | 74 | 33 | 14.3 | 14.4 | 10.9 | 70 | 14.2 | – | 3.3 | – | 13.3 | 85 | 184 | 10.5 | 146 | 5.7 | 2.4 | 0.5 | – | – | 15.9 | 281 | 40 | 60 | 64 | 75 | 4.5 | 54 | 14.5 | 7.2 | 2.5 |
|  | 5-10 | 1206 | 98 | 39 | 9.8 | 16.3 | 8.2 | 47 | 19.9 | 0.7 | 5.2 | – | 14.9 | 112 | 240 | 15.1 | 214 | 8.7 | 2.2 | 0.2 | – | – | 11.1 | 320 | 34 | 60 | 50 | 65 | 6.9 | 11 | 19.5 | 10.1 | 3.4 |
|  | 10-20 | 735 | 106 | 42 | 10.5 | 15.1 | 6.0 | 46 | 20.6 | 1.1 | 5.6 | 0.3 | 18.0 | 112 | 257 | 16.7 | 228 | 9.1 | 2.0 | – | 1.3 | – | 13.3 | 314 | 39 | 66 | 55 | 76 | 6.9 | 5 | 20.9 | 11.2 | 4.4 |
|  | 20–30 | 613 | 108 | 45 | 9.8 | 15.9 | 6.2 | 50 | 20.9 | 0.8 | 5.8 | 0.2 | 19.3 | 115 | 259 | 17.8 | 226 | 10.1 | 1.7 | – | – | – | 11.4 | 319 | 36 | 63 | 51 | 67 | 6.3 | 6 | 23.3 | 12.3 | 4.2 |
| **Slope 4** | | | | | |  |  |  |  |  |  |  |  |  |  |  |  |  |  |  |  |  |  |  |  |  |  |  |  |  |  |  |  |
| **L2-S2-P1-1** | 0-5 | 1883 | 84 | 35 | 12.2 | 15.6 | 9.1 | 56 | 17.2 | 0.7 | 4.3 | – | 11.4 | 110 | 200 | 12.5 | 169 | 7.4 | 2.6 | 0.3 | – | – | 13.1 | 337 | 34 | 56 | 54 | 63 | 5.5 | 29 | 15.4 | 8.8 | 3.8 |
|  | 5-10 | 1230 | 93 | 37 | 8.1 | 15.8 | 7.6 | 51 | 18.4 | 0.9 | 4.1 | – | 12.3 | 118 | 205 | 13.1 | 183 | 8.6 | 2.1 | 0.3 | – | – | 11.0 | 359 | 33 | 55 | 49 | 66 | 4.8 | 12 | 17.1 | 9.3 | 3.4 |
|  | 10-20 | 472 | 105 | 43 | 6.5 | 15.9 | 6.5 | 53 | 20.9 | 0.9 | 5.6 | – | 10.8 | 127 | 211 | 15.6 | 203 | 9.4 | 1.7 | – | – | – | 11.0 | 401 | 34 | 60 | 43 | 67 | 6.6 | 8 | 19.1 | 11.0 | 4.1 |
| **L2-S2-P1-2** | 0-5 | 1922 | 84 | 33 | 10.3 | 13.3 | 5.7 | 50 | 16.1 | 0.8 | 3.5 | 0.3 | 12.9 | 105 | 198 | 12.1 | 165 | 7.1 | 2.4 | 0.4 | 1.2 | – | 15.3 | 330 | 40 | 63 | 67 | 81 | 6.1 | 33 | 15.0 | 7.9 | 3.1 |
|  | 5-10 | 1266 | 93 | 37 | 11.9 | 16.0 | 5.9 | 51 | 18.0 | 0.9 | 4.1 | – | 11.2 | 115 | 201 | 13.0 | 183 | 7.9 | 2.1 | – | – | – | 10.8 | 355 | 32 | 55 | 48 | 64 | 5.8 | 16 | 16.1 | 8.7 | 3.6 |
|  | 10-20 | 456 | 105 | 42 | 5.0 | 15.9 | 6.7 | 51 | 20.7 | 1.0 | 5.3 | – | 10.8 | 131 | 217 | 15.1 | 211 | 9.3 | 2.0 | – | 1.7 | – | 12.6 | 387 | 35 | 59 | 53 | 74 | 6.3 | 6 | 18.9 | 10.3 | 4.1 |
| **L2-S2-P2-1** | 0-5 | 1889 | 87 | 36 | 11.0 | 16.5 | 8.7 | 54 | 17.2 | 0.8 | 3.6 | – | 12.2 | 110 | 207 | 13.1 | 176 | 7.4 | 1.5 | 0.2 | – | – | 9.5 | 337 | 31 | 52 | 43 | 57 | 5.0 | 31 | 16.3 | 9.2 | 3.6 |
|  | 5-10 | 1221 | 99 | 38 | 8.8 | 15.2 | 7.1 | 53 | 19.3 | 0.7 | 4.5 | – | 12.5 | 123 | 213 | 14.2 | 183 | 8.1 | 1.9 | – | – | – | 12.1 | 370 | 34 | 58 | 48 | 66 | 5.6 | 11 | 17.3 | 9.7 | 4.0 |
|  | 10-20 | 543 | 110 | 43 | 8.3 | 16.2 | 7.0 | 54 | 20.9 | 1.0 | 5.1 | – | 13.8 | 133 | 223 | 16.5 | 209 | 9.3 | 1.0 | – | – | – | 9.6 | 405 | 34 | 61 | 48 | 63 | 7.2 | 7 | 19.7 | 11.0 | 4.1 |
| **L2-S2-P2-2** | 0-5 | 1588 | 91 | 36 | 10.4 | 16.0 | 10.3 | 53 | 18.8 | 0.8 | 3.7 | – | 12.3 | 117 | 208 | 13.5 | 181 | 8.1 | 1.7 | – | – | – | 8.5 | 346 | 29 | 50 | 45 | 54 | 4.6 | 29 | 17.5 | 9.2 | 3.8 |
|  | 5-10 | 913 | 105 | 41 | 5.3 | 13.6 | 5.6 | 49 | 19.5 | 0.8 | 4.4 | – | 12.4 | 128 | 216 | 15.0 | 205 | 8.8 | 2.2 | – | – | – | 10.8 | 391 | 33 | 56 | 47 | 60 | 6.6 | 16 | 18.4 | 9.6 | 3.7 |
|  | 10-20 | 448 | 112 | 45 | 10.7 | 14.7 | 6.8 | 50 | 21.9 | 0.9 | 5.2 | – | 15.7 | 138 | 233 | 16.8 | 203 | 9.4 | 2.4 | – | – | – | 8.6 | 389 | 30 | 56 | 36 | 57 | 6.4 | 8 | 20.8 | 11.4 | 5.1 |

*ppm = parts per million

Gerald Raab^a,b*^, Markus Egli^a^, Kevin P. Norton^c^, Adam P. Martin^d^, Michael E. Ketterer^e^, Dmitry Tikhomirov^a^, Rahel Wanner^f^, Fabio Scarciglia^g^

^a^ Department of Geography, University of Zurich, Winterthurerstrasse 190, 8057 Zurich, Switzerland

^b^ Department of Earth and Environmental Sciences, Dalhousie University, PO BOX 15000, 1459 Oxford Street, Halifax

^c^ School of Geography, Environment and Earth Sciences, Te Herenga Waka, Victoria University of Wellington, PO Box 600, 6140 Wellington, New Zealand

^d^ GNS Science, Private Bag 1930, Dunedin, New Zealand

^e^ Chemistry and Biochemistry, Northern Arizona University, Box 5698, Flagstaff, AZ 86011-5698, USA

^f^ Institute of Natural Resource Sciences, Zurich University of Applied Sciences, Grüental, 8820 Wädenswil, Switzerland

^g^ Department of Biology, Ecology and Earth Sciences (DiBEST), University of Calabria, Via P. Bucci – Cubo 15B, 87036 Arcavacata di Rende (CS), Italy

*Corresponding author. Tel.: +41 44 635 65 27; Fax: +41 44 6356848.

E-mail address: gr.science@gmx.at (G. Raab).
