# Supplementary material for: Soil weathering dynamics and erosion in a dry oceanic area of the southern hemisphere (Otago, New Zealand)
Source: Sci Rep. 2022 Nov 17;12:19803. doi: 10.1038/s41598-022-23731-7 (PMC9672066; doi:10.1038/s41598-022-23731-7)
Supplement: Supplementary file 9 — Supplementary Table S4. [file 41598_2022_23731_MOESM9_ESM.doc]

**Table S4**: Overview of the tested weathering indices.

| **Weathering Index** | **Equation** | **Reference** |
| --- | --- | --- |
| Vogt ratio | 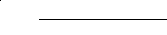 | Vogt (1927) |
| Silico-aluminum ratio | 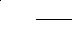 | Ruxton (1968) |
| Weathering index of Parker | 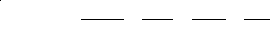 | Parker (1970)  Price & Velbel (2003) |
| Index B | 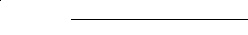 | Kronberg & Nesbitt (1981) |
| Chemical index of alteration | 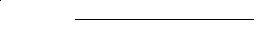 | Nesbitt & Young (1982) |
| Chemical index of weathering | 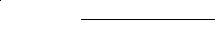 | Harnois (1988) |
| Plagioclase index of alteration | 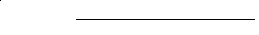 | Fedo et al. (1995) |
| Chemical proxy of alteration | 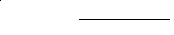 | Buggle et al. (2011) |
| Potassium, calcium and titanium ratio | 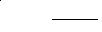 | Egli et al. (2008)  Raab et al. (2017) |

Gerald Raab^a,b*^, Markus Egli^a^, Kevin P. Norton^c^, Adam P. Martin^d^, Michael E. Ketterer^e^, Dmitry Tikhomirov^a^, Rahel Wanner^f^, Fabio Scarciglia^g^

^a^ Department of Geography, University of Zurich, Winterthurerstrasse 190, 8057 Zurich, Switzerland

^b^ Department of Earth and Environmental Sciences, Dalhousie University, PO BOX 15000, 1459 Oxford Street, Halifax

^c^ School of Geography, Environment and Earth Sciences, Te Herenga Waka, Victoria University of Wellington, PO Box 600, 6140 Wellington, New Zealand

^d^ GNS Science, Private Bag 1930, Dunedin, New Zealand

^e^ Chemistry and Biochemistry, Northern Arizona University, Box 5698, Flagstaff, AZ 86011-5698, USA

^f^ Institute of Natural Resource Sciences, Zurich University of Applied Sciences, Grüental, 8820 Wädenswil, Switzerland

^g^ Department of Biology, Ecology and Earth Sciences (DiBEST), University of Calabria, Via P. Bucci – Cubo 15B, 87036 Arcavacata di Rende (CS), Italy

*Corresponding author. Tel.: +41 44 635 65 27; Fax: +41 44 6356848.

E-mail address: gr.science@gmx.at (G. Raab).
