# Supplementary material for: Soil weathering dynamics and erosion in a dry oceanic area of the southern hemisphere (Otago, New Zealand)
Source: Sci Rep. 2022 Nov 17;12:19803. doi: 10.1038/s41598-022-23731-7 (PMC9672066; doi:10.1038/s41598-022-23731-7)
Supplement: Supplementary file 10 — Supplementary Table S5. [file 41598_2022_23731_MOESM10_ESM.docx]

**Table S5**: Trace elemental content of measured rock samples along tors. The blue shaded samples indicate at the ridge location Tor 2, and for the valley location Tor 1, which are each closest to the corresponding soil sampling sites.

| Sample position | Sampling Material | S | V | Cr | Co | Ni | Cu | Zn | Ga | Ge | As | Se | Br | Rb | Sr | Y | Zr |
| --- | --- | --- | --- | --- | --- | --- | --- | --- | --- | --- | --- | --- | --- | --- | --- | --- | --- |
|  |  | *ppm | *ppm | *ppm | *ppm | *ppm | *ppm | *ppm | *ppm | *ppm | *ppm | *ppm | *ppm | *ppm | *ppm | *ppm | *ppm |
| Ridge | Schist | 113 | 63 | 111 | – | 12.9 | 12.4 | 52 | 13.8 | – | 1.5 | – | 0.6 | 88 | 164 | 14.7 | 129 |
| Ridge | Schist | 76 | 59 | 60 | 12.5 | 19.7 | 19.1 | 65 | 18.0 | 0.5 | 6.8 | – | 0.9 | 141 | 232 | 12.8 | 160 |
| Ridge | Schist | 83 | 74 | 129 | 4.5 | 12.2 | 19.9 | 41 | 18.7 | 0.5 | 14.3 | – | 0.3 | 99 | 160 | 12.2 | 216 |
| Ridge | Schist | 39 | 94 | 140 | 10.8 | 21.0 | 10.5 | 70 | 2- | 0.5 | 6.6 | – | 1.2 | 137 | 303 | 21.8 | 239 |
| Ridge | Schist | 113 | 102 | 58 | 8.9 | 21.5 | 22.2 | 77 | 20.8 | 0.6 | 4.7 | – | 0.3 | 111 | 292 | 21.3 | 163 |
| Ridge | Schist | 128 | 115 | 68 | 9.3 | 25.4 | 28.6 | 92 | 22.9 | 0.7 | 3.1 | – | 1.2 | 151 | 306 | 29.3 | 166 |
| Ridge | Schist | 154 | 17 | 44 | – | 9.5 | 14.3 | 20 | 9.8 | – | 0.9 | 0.1 | 1.0 | 20 | 78 | 3.3 | 21 |
| Ridge | Schist | 37 | 135 | 98 | 10.2 | 23.5 | 26.4 | 86 | 23.7 | 0.4 | 3.5 | – | 0.3 | 171 | 342 | 27.1 | 207 |
| Ridge | Schist | 41 | 57 | 128 | 9.6 | 19.5 | 16.4 | 51 | 15.1 | 0.3 | 2.8 | – | 0.3 | 75 | 165 | 13.8 | 109 |
| Ridge | Schist | 27 | 79 | 111 | 15.3 | 19.9 | 5.3 | 57 | 18.2 | 0.4 | 6.0 | – | 1.2 | 102 | 247 | 18.2 | 196 |
| Ridge | Schist | 101 | 104 | 82 | 11.9 | 24.7 | 20.5 | 78 | 2– | 0.5 | 9.0 | – | – | 142 | 313 | 23.4 | 174 |
| Ridge | Schist | 82 | 82 | 104 | 8.6 | 18.4 | 16.7 | 59 | 19.0 | 1.0 | 7.7 | – | 0.5 | 112 | 284 | 18.1 | 191 |
| Ridge | Schist | 76 | 86 | 121 | 4.7 | 17.7 | 14.3 | 58 | 17.4 | 0.4 | 5.8 | – | 0.4 | 103 | 270 | 20.3 | 191 |
| Ridge | Schist | 50 | 104 | 82 | 10.3 | 24.4 | 13.5 | 81 | 21.8 | 0.6 | 9.4 | – | 0.4 | 135 | 357 | 25.5 | 223 |
| Ridge | Schist | 64 | 101 | 94 | 9.9 | 19.7 | 21.9 | 73 | 22.8 | 0.6 | 9.7 | – | 1.0 | 151 | 344 | 20.1 | 230 |
| Ridge | Schist | 287 | 147 | 97 | 13.3 | 26.9 | 38.3 | 100 | 24.8 | 0.5 | 5.2 | – | 0.5 | 170 | 325 | 30.6 | 162 |
| Ridge | Schist | 92 | 133 | 79 | 15.9 | 25.6 | 26.8 | 100 | 25.5 | 0.5 | 3.9 | – | 0.4 | 177 | 283 | 28.6 | 200 |
| Ridge | Schist | 29 | 146 | 83 | 18.3 | 28.0 | 28.3 | 100 | 24.6 | 0.7 | 5.7 | – | 0.3 | 166 | 300 | 29.4 | 194 |
| Ridge | Schist | 37 | 122 | 82 | 17.6 | 28.6 | 19.6 | 89 | 22.9 | 0.4 | 6.6 | – | 0.8 | 129 | 263 | 25.4 | 225 |
| Valley | Schist | 58 | 110 | 72 | 9.3 | 25.5 | 16.7 | 91 | 23.7 | 1.1 | 7.6 | – | 0.3 | 148 | 320 | 39.6 | 268 |
| Valley | Schist | 69 | 87 | 61 | 15.5 | 18.3 | 10.2 | 63 | 16.3 | 0.4 | 4.6 | – | 0.3 | 60 | 169 | 21.1 | 143 |
| Valley | Schist | 88 | 101 | 70 | 17.6 | 18.2 | 14.4 | 79 | 18.5 | 0.8 | 5.0 | – | 0.7 | 92 | 188 | 26.4 | 190 |
| Valley | Schist | 27 | 143 | 91 | 18.6 | 30.3 | 29.1 | 111 | 22.0 | 0.4 | 7.1 | – | 0.7 | 119 | 232 | 29.5 | 181 |
| Valley | Schist | 264 | 134 | 93 | 8.1 | 21.8 | 23.7 | 92 | 23.9 | 0.6 | 3.9 | – | 0.2 | 147 | 226 | 26.9 | 196 |
| Valley | Schist | 90 | 140 | 87 | 9.6 | 32.8 | 32.3 | 111 | 28.6 | 0.9 | 5.0 | – | 0.5 | 189 | 127 | 10.6 | 241 |
| Valley | Schist | 39 | 121 | 96 | 11.0 | 37.3 | 47.0 | 116 | 25.1 | 0.7 | 9.2 | – | 1.4 | 150 | 82 | 8.3 | 216 |
| Valley | Schist | 539 | 38 | 229 | – | 9.4 | 8.7 | 39 | 12.0 | 0.3 | 0.4 | – | 0.2 | 62 | 140 | 9.1 | 82 |
| Valley | Schist | 124 | 47 | 147 | – | 8.9 | 12.5 | 39 | 13.5 | 0.3 | 0.4 | – | 0.3 | 65 | 236 | 12.3 | 139 |
| Valley | Schist | 78 | 53 | 80 | – | 12.3 | 9.1 | 49 | 17.3 | 0.4 | 1.6 | – | 0.3 | 95 | 247 | 14.7 | 146 |

| Sample position | Sampling Material | Nb | Mo | Cd | Sn | Cs | Ba | La | Ce | Pr | Nd | Hf | W | Pb | Th | U |
| --- | --- | --- | --- | --- | --- | --- | --- | --- | --- | --- | --- | --- | --- | --- | --- | --- |
|  |  | *ppm | *ppm | *ppm | *ppm | *ppm | *ppm | *ppm | *ppm | *ppm | *ppm | *ppm | *ppm | *ppm | *ppm | *ppm |
| Ridge | Schist | 5.7 | 7.0 | – | – | 6.7 | 385 | 29 | 55 | 38 | 59 | 4.0 | – | 11.8 | 9.5 | 2.7 |
| Ridge | Schist | 6.1 | 3.7 | – | – | 6.6 | 483 | 23 | 46 | 28 | 43 | 3.3 | 0.4 | 21.9 | 13.2 | 4.7 |
| Ridge | Schist | 7.9 | 8.4 | – | – | 5.9 | 616 | 32 | 59 | 42 | 62 | 4.9 | – | 32.2 | 16.2 | 3.7 |
| Ridge | Schist | 8.0 | 7.1 | 0.2 | 1.3 | 13.7 | 593 | 44 | 77 | 54 | 73 | 7.0 | 0.7 | 26.4 | 16.1 | 5.9 |
| Ridge | Schist | 8.9 | 1.9 | – | 0.8 | 6.1 | 441 | 36 | 69 | 36 | 64 | 5.1 | 1.3 | 21.3 | 13.7 | 4.5 |
| Ridge | Schist | 10.0 | 2.8 | – | – | 4.9 | 617 | 38 | 75 | 33 | 67 | 5.8 | 1.5 | 29.8 | 17.8 | 5.3 |
| Ridge | Schist | 0.6 | 3.5 | – | – | 2.9 | 94 | 14 | 26 | 31 | 36 | – | – | 20.0 | 2.2 | – |
| Ridge | Schist | 10.6 | 3.5 | 0.2 | – | 7.3 | 659 | 39 | 76 | 37 | 64 | 8.1 | 1.6 | 31.7 | 20.0 | 6.0 |
| Ridge | Schist | 4.7 | 5.9 | – | – | 6.2 | 331 | 28 | 49 | 40 | 51 | 2.2 | 0.4 | 22.6 | 8.7 | 2.2 |
| Ridge | Schist | 6.7 | 4.7 | – | – | 9.5 | 461 | 37 | 68 | 46 | 73 | 5.6 | 0.9 | 20.1 | 13.8 | 4.7 |
| Ridge | Schist | 8.9 | 3.5 | – | – | 14.0 | 561 | 47 | 81 | 54 | 77 | 5.4 | 1.5 | 20.5 | 16.6 | 5.3 |
| Ridge | Schist | 8.6 | 5.8 | – | 1.2 | 9.6 | 506 | 34 | 65 | 36 | 56 | 5.7 | 0.7 | 24.1 | 15.6 | 5.4 |
| Ridge | Schist | 9.3 | 6.8 | – | – | 11.6 | 469 | 39 | 76 | 48 | 74 | 5.5 | 0.3 | 24.1 | 16.0 | 4.8 |
| Ridge | Schist | 10.8 | 4.1 | 0.2 | 2.3 | 15.8 | 569 | 51 | 91 | 64 | 91 | 5.7 | 0.9 | 26.5 | 18.4 | 6.3 |
| Ridge | Schist | 10.8 | 4.8 | – | – | 13.2 | 637 | 43 | 80 | 51 | 74 | 5.6 | – | 35.1 | 18.2 | 5.9 |
| Ridge | Schist | 12.8 | 3.6 | – | 1.0 | 7.8 | 626 | 41 | 81 | 36 | 72 | 7.0 | 1.1 | 28.1 | 19.6 | 6.0 |
| Ridge | Schist | 11.3 | 2.1 | – | 0.8 | 5.6 | 648 | 38 | 79 | 30 | 61 | 7.4 | 1.8 | 24.2 | 19.2 | 6.6 |
| Ridge | Schist | 12.3 | 2.3 | – | – | 9.6 | 587 | 47 | 88 | 49 | 72 | 7.8 | 1.6 | 30.7 | 20.6 | 5.2 |
| Ridge | Schist | 11.3 | 3.8 | – | 1.4 | 7.2 | 452 | 41 | 76 | 40 | 61 | 7.2 | 2.0 | 28.9 | 18.8 | 5.2 |
| Valley | Schist | 9.8 | 2.5 | – | 1.4 | 8.6 | 684 | 42 | 85 | 49 | 85 | 10.9 | 1.8 | 25.6 | 18.3 | 6.9 |
| Valley | Schist | 6.3 | 2.7 | – | – | 3.9 | 289 | 27 | 56 | 33 | 50 | 3.5 | 0.6 | 16.3 | 9.5 | 1.8 |
| Valley | Schist | 7.6 | 2.8 | – | – | 3.7 | 411 | 28 | 58 | 27 | 52 | 6.0 | 0.8 | 21.7 | 11.6 | 3.1 |
| Valley | Schist | 9.8 | 3.2 | – | 1.9 | 7.8 | 500 | 36 | 77 | 34 | 66 | 7.4 | 1.7 | 24.2 | 15.0 | 3.5 |
| Valley | Schist | 10.4 | 4.4 | – | 1.0 | 9.7 | 592 | 43 | 75 | 46 | 67 | 6.6 | 1.6 | 29.5 | 15.8 | 4.0 |
| Valley | Schist | 13.2 | 2.5 | 0.6 | 3.6 | 19.6 | 784 | 41 | 64 | 76 | 92 | 8.1 | 1.7 | 22.7 | 18.7 | 3.1 |
| Valley | Schist | 12.4 | 4.5 | – | – | 9.1 | 577 | 23 | 42 | 46 | 53 | 7.0 | 0.5 | 29.7 | 16.5 | 1.2 |
| Valley | Schist | 3.2 | 13.2 | 0.2 | – | 4.8 | 352 | 19 | 37 | 32 | 47 | 1.7 | – | 21.9 | 5.1 | 0.7 |
| Valley | Schist | 4.4 | 8.1 | – | – | 3.9 | 388 | 23 | 47 | 28 | 44 | 2.9 | – | 14.1 | 7.8 | 2.6 |
| Valley | Schist | 5.2 | 4.8 | – | – | 4.7 | 617 | 27 | 51 | 35 | 50 | 3.3 | 0.5 | 16.9 | 9.9 | 3.6 |

*ppm = parts per million

Gerald Raab^a,b*^, Markus Egli^a^, Kevin P. Norton^c^, Adam P. Martin^d^, Michael E. Ketterer^e^, Dmitry Tikhomirov^a^, Rahel Wanner^f^, Fabio Scarciglia^g^

^a^ Department of Geography, University of Zurich, Winterthurerstrasse 190, 8057 Zurich, Switzerland

^b^ Department of Earth and Environmental Sciences, Dalhousie University, PO BOX 15000, 1459 Oxford Street, Halifax

^c^ School of Geography, Environment and Earth Sciences, Te Herenga Waka, Victoria University of Wellington, PO Box 600, 6140 Wellington, New Zealand

^d^ GNS Science, Private Bag 1930, Dunedin, New Zealand

^e^ Chemistry and Biochemistry, Northern Arizona University, Box 5698, Flagstaff, AZ 86011-5698, USA

^f^ Institute of Natural Resource Sciences, Zurich University of Applied Sciences, Grüental, 8820 Wädenswil, Switzerland

^g^ Department of Biology, Ecology and Earth Sciences (DiBEST), University of Calabria, Via P. Bucci – Cubo 15B, 87036 Arcavacata di Rende (CS), Italy

*Corresponding author. Tel.: +41 44 635 65 27; Fax: +41 44 6356848.

E-mail address: gr.science@gmx.at (G. Raab).
